# Supplementary material for: Defining and identifying critical elements of, and lessons learned from addressing, ‘operational readiness’ for public health emergency events, including COVID-19: a scoping review protocol
Source: BMJ Open. 2022 Sep 19;12(9):e060526. doi: 10.1136/bmjopen-2021-060526 (PMC9485644; doi:10.1136/bmjopen-2021-060526)
Supplement: Author's manuscript [file bmjopen-2021-060526.draft_revisions.pdf]

BMJ Open is committed to open peer review. As part of this commitment we make the peer review history of every article we publish publicly available.

When an article is published we post the peer reviewers' comments and the authors' responses online. We also post the versions of the paper that were used during peer review. These are the versions that the peer review comments apply to.

The versions of the paper that follow are the versions that were submitted during the peer review process. They are not the versions of record or the final published versions. They should not be cited or distributed as the published version of this manuscript.

BMJ Open is an open access journal and the full, final, typeset and author-corrected version of record of the manuscript is available on our site with no access controls, subscription charges or pay-per-view fees (http://bmjopen.bmj.com).

If you have any questions on BMJ Open's open peer review process please email info.bmjopen@bmj.com

BMJ Open

# Defining and identifying critical elements of, and lessons learned from addressing, 'operational readiness' for public health emergency events, including COVID-19: a rapid scoping review protocol

| | |
|---|---|
| Journal: | *BMJ Open* |
| Manuscript ID | bmjopen-2021-060526 |
| Article Type: | Protocol |
| Date Submitted by the Author: | 17-Jan-2022 |
| Complete List of Authors: | English, Rene; Stellenbosch University Faculty of Medicine and Health Sciences, Global Health<br>Geduld, Heike; Stellenbosch University, . Division of Emergency Medicine, Department of Family and Emergency Medicine, Faculty of Medicine and Health Sciences<br>Louw, QA; Stellenbosch University, Physiotherapy; Stellenbosch University,<br>McCaul, Michael; Stellenbosch University Faculty of Medicine and Health Sciences, Department of Interdisciplinary Sciences; Stellenbosch<br>Joseph, Conran; Stellenbosch University, Division of Physiotherapy, Department of Health and Rehabilitation Sciences,<br>Pappin, Michele; Stellenbosch University, Faculty of Medicine and Health Sciences, Global Health Faculty of Medicine and Health Sciences Division of Health Systems and Public Health<br>Berner, Karina; Stellenbosch University Faculty of Medicine and Health Sciences, Rehabilitation Sciences<br>Nyasulu, Juliet; Stellenbosch University, Faculty of Medicine and Health Sciences, Global Health Faculty of Medicine and Health Sciences Division of Health Systems and Public Health; AFRIQUIP, Health Systems Strengthening |
| Keywords: | INFECTIOUS DISEASES, PUBLIC HEALTH, ACCIDENT & EMERGENCY MEDICINE |
| | |

SCHOLARONE™
Manuscripts

# TITLE: Defining and identifying critical elements of, and lessons learned from addressing, 'operational readiness' for public health emergency events, including COVID-19: a rapid scoping review protocol


## Abstract

**Introduction:** 'Readiness' comprises the immediate actions needed to respond to a developing risk or hazard. Knowledge about critical readiness components and actions required by countries at all levels in response to health emergencies is critical to inform operational readiness actions for future events.

**Objective:** To define and identify the critical elements of 'operational readiness' for public health emergencies, including COVID-19, and identify lessons learnt from addressing it, to inform the World Health Organisation (WHO) Operational Readiness Framework.

**Methods:** Review will follow Joanna Briggs Institute guidance. Reporting will be done using relevant PRISMA Extensions. MEDLINE, Embase and Web of Science databases will be searched. Grey literature includes targeted repositories, websites, and databases of organisations involved in health emergencies and disaster risk management. Searches will be limited to 2010–2021 and English availability. Twenty percent of titles and abstracts will be screened by two reviewers. One reviewer will subsequently screen remaining titles and abstracts while the second will verify exclusions. Potentially eligible full texts will be screened by one reviewer, while a second will verify exclusions. Disagreements will be resolved through discussion. Depending on the yield of included sources, data extraction will be done using a dynamic approach involving one or more reviewers and an additional verifying reviewer. A pilot-tested custom extraction form will be used. Uncertainties will be discussed and and included documents will be analysed by two reviewers using qualitative thematic analysis through a deductive synthesis approach.

**Dissemination:** By defining evidence related to critical readiness components and actions, the review will reveal new insights, knowledge and lessons learnt that will translate into an operational framework for readiness actions. In consultation with WHO, findings will be disseminated as appropriate (e.g., through professional bodies, conferences, and research papers).

**Protocol registration:** This rapid scoping review has been registered on Open Science Framework (doi:10.17605/OSF.IO/6SYAH).



**Key Words:** Operational readiness, Public health, emergency events, Health systems, Rapid scoping review

**Number of words:** 3216


For peer review only

**Strengths and limitations for the scoping review**

- The Covid-19 pandemic has shown that globally, countries even with well-resourced health systems and structured emergency preparedness plans in place were not able to sufficiently respond to the threat. Meaning that gaps existed between transition from preparedness to responding which is readiness. Therefore, defining and identifying critical elements of operational readiness for public health emergency events, including COVID-19 is critical
- Emergency operational readiness is embedded between preparedness and response and in most cases poorly defined. Therefore, we believe that an understanding of health systems readiness in responding to emergency is key.
- Currently there is no clear definition of activities that constitute health systems emergency readiness and people use different names whilst others name it either preparedness or response. In case these operational readiness definition words are not captured in the scoping review search strategy, this will be a limitation.

## Introduction

Much has been documented about how countries should best prepare to respond to health emergencies (1–3). The effectiveness of 'readiness' – a concept referring to actions needed to rapidly respond to an imminently anticipated risk or hazard – largely depends on the sufficiency and comprehensiveness of prior longer-term 'preparedness' policies (4). However, little is known about the critical components of readiness and the kinds of readiness actions that should be taken by countries at all levels in response to health emergencies. Such knowledge is critical to inform operational readiness actions for future events.

Health Emergencies and Disaster Risk Management (Health-EDRM) encompasses the intersecting fields of emergency and disaster medicine, health systems strengthening and resilience, disaster risk reduction, humanitarian response and community health resilience. Within this framework, it is accepted that the management of emergencies is a whole-of-society approach, focusing on all hazards and involving multiple sectors and multiple disciplines (5). Health-EDRM involves four broad components, namely (i) hazard vulnerability assessment (HVA) and mitigation; (ii) preparedness; (iii) response and (iv) recovery. Within these, the activities of 'readiness' will occur within both HVA and mitigation and preparedness components. These readiness activities are linked both temporally and structurally to a specifically identified hazard, whether that is an infectious disease, or climate change event. Thus, what constitutes 'readiness' is determined by the nature of the hazard.

The World Health Organisation (WHO) Strategic Framework for Emergency Preparedness (6) is a unifying framework for country-level public health emergency preparedness. This framework describes operational readiness to respond to emergencies as a continuous, co-ordinated process, involving a multisectoral response, incorporating multiple level infrastructure, and following an all-hazard approach with a focus on high priority risks (6).

For peer review only

The current COVID-19 global pandemic has exposed the fragility of health systems to respond to shocks in the form of disease outbreaks or health emergencies (7). According to the WHO, the response of a public health system to an outbreak or health emergency such as the COVID-19 pandemic can be defined as a cycle that sways between preparedness and the actual response. Through applying a governance lens, the WHO has developed an Emergency Response Framework (4), which describes the stages of an outbreak or health emergency. As alluded to above, readiness to respond lies somewhere between preparedness and response; it is the instant action to an emergent or prominent risk and is hugely reliant on adequate preparedness (4). In many instances, implementation of these well-designed disaster preparedness policies is met with significant challenges due to flaws in the 'readiness' of systems to do so. 'Readiness' as a concept has not been fully designed, and therefore it is critical to define the critical components of readiness and the types of readiness actions to be taken in response to outbreaks and health emergencies to inform operational readiness actions for future events (8). A preliminary search of MEDLINE, the Cochrane Database of Systematic Reviews, Prospero and JBI Evidence Synthesis revealed no current or underway systematic reviews or scoping reviews on the topic. The WHO is currently developing an Operational Readiness Framework intended to guide effective action. Specifically, the purpose of the framework is to scale-up preparedness for a specific risk at the local and national levels by considering how ready a country is to respond to the imminent threat, and to identify key actions needed to be ready to respond effectively to that threat. To this end, WHO has called for a rapid scoping review to be conducted that will assist with defining available evidence related to readiness and readiness actions.

## Aim and objectives

The overarching aim of this rapid scoping review is to define and identify the critical elements of 'operational readiness' for public health emergencies, including COVID-19, and identify lessons learnt from addressing it, to inform the WHO Operational Readiness Framework.

To this end, the following objectives will be addressed:

1. To conceptualise and define 'operational readiness';
2. To map and describe frameworks, policies and evidence/information related to 'operational readiness' for all hazards, with a strong focus on infectious diseases;
3. To define critical elements of 'operational readiness' at multiple levels of the health system (community, local, sub-national, national, regional, global);
4. To identify lessons learned from enhancing or influencing 'operational readiness' (at multiple levels).

For peer review only

# Review question

## Primary scoping review method question

The primary review question was formulated using the PCC (Population, Concept and Context) method (9): *How can/do communities/countries/regions/global institutions operationalise readiness for imminent public emergencies?*

## Sub-questions

The review will seek to answer the following additional or sub-questions:

1. How is 'operational readiness' for public health emergencies conceptualised and defined?
2. What are the critical elements (dimensions, operational actions, coordination) of 'operational readiness' for public health emergencies at multiple levels (community, local, sub-national, national, regional, global)?
3. How did countries ready/ prepare for COVID-19?
4. What lessons have been learned about 'operational readiness' during for example, COVID-19/ Ebola, with a strong focus on infectious disease emergencies?

# Keywords

All hazards; Disaster planning; Epidemic; Imminent threat; Infectious diseases; Outbreak; Pandemic; Public health emergency

# Eligibility criteria

## Inclusion criteria

### *Participants/ population*

These are the groups or organisations who would respond and/or lead the response, and include the following:

- Communities (local, subregional or national level)
- National, country, regional and global governments
- Global health organisations, such as the WHO

For peer review only

### *Concept*

The purpose of the scoping review is to define 'operational readiness. This concept refers to the immediate action(s) that are taken to pre-position response actions needed to address a proximal, imminent hazard/ threat – such as an 'acute' infectious disease outbreak or natural disaster threat (an all-hazards approach). The concept lies between 'preparedness' and 'response'. To find evidence of readiness interventions, we will look at sources referencing preparedness, planning and disaster management as the term 'readiness' may be embedded in 'preparedness' – or the term 'preparedness' may be used to describe actions that (based on our definition) we would describe as readiness.

We will consider sources that:

- Conceptualise, theorise, define, or describe or interpret 'operational readiness' and/or preparedness for public health emergencies (at community, country, regional or global levels) at the time when the threat of an infectious disease outbreaks or natural disaster becomes known, within a specific timeframe (viz., defining 'imminence');
- Contain explanations, descriptions, intervention approaches, analysis or frameworks or anticipatory actions for 'operational readiness' or preparedness for public health emergencies (at community, country, regional or global levels) when the threat of an infectious disease outbreaks or natural disaster becomes known;
- Provide the nature and description of critical elements (dimensions, coordination, roles of key stakeholders such as the community, health actors, policy makers etc.) of 'operational readiness' for public health emergencies at community, national, regional, and global levels.

### *Context*

The context of health emergencies refers to natural disasters and infectious disease threats (new and re-emerging) – i.e., all hazards. Important to note is that these threats are acute (imminent) and impact the health of populations. These health emergencies occur within the community as well as health system and health service contexts.

The proposed definition of a 'health emergency' is an extraordinary event that is determined to 'constitute a public health risk whose scale, timing, or unpredictability threatens to overwhelm routine capabilities of the health system' (10 pS9) and potentially require a coordinated response at multiple levels (10,11).

### *Types of* sources

- Peer-reviewed review or empirical research (any study design) that is available in full-text and published in scientific journals between 2010 and 2021;
- Publicly available policy frameworks and programme reports;
  Published conference reports or electronic theses;

For peer review only

- Documents of which the full text or abstract is available in the English language. If the English version of the abstract is potentially eligible for inclusion, the full text (if German/ French/ Afrikaans) will be translated to make a final decision on eligibility.

### Exclusion criteria

- Papers focusing exclusively on longer term preparedness actions or exclusively on response actions will be excluded;
- Papers reporting on contexts beyond health emergencies or not focused on disease prevention and control will be excluded.

## Methods

This rapid scoping review will be conducted in accordance with the Joanna Briggs Institute (JBI) methodology for scoping reviews (9). The review will be reported using the PRISMA Extension for Scoping Reviews (PRISMA-ScR) (12) and PRISMA-S Extension for Searches in Systematic Reviews (13).

### Search strategy

The search strategy will aim to locate peer-reviewed review or empirical research (any study design) that is available in full-text and published in scientific journals, publicly available policy frameworks, programme reports, and published conference reports or electronic theses. This will include humanitarian literature where health impacts or effects are the focus. Due to the rapid nature of the scoping review, we will restrict the search to studies published between 2010 – 2021 and those available in English (potentially eligible Afrikaans, German or French full texts, according to the English abstract, will be translated into English).

The electronic databases to be searched include MEDLINE, Embase and Web of Science. An initial limited search of MEDLINE was undertaken to identify articles on the review topic. The text words contained in the titles and abstracts of relevant articles, and the index terms used to describe the articles were used to draft a full search strategy for MEDLINE. The search strings and terms were developed iteratively and in consultation with the WHO and are centred around three key concepts: (i) emergencies/ diseases/ natural disasters; (ii) readiness/ preparedness/ risk/ planning; and (iii) health systems/ community. The search strategy, including all identified keywords and index terms, was subsequently adapted for Embase and Web of Science (see Appendix I). Searches will be conducted by an expert information specialist in consultation with the review team. The reference list of all included sources of evidence will also be screened for additional studies. Reporting of the searching will be guided by the PRISMA-S Extension for Searches in Systematic Reviews (13).

#### *Searching other resources*

Sources of unpublished studies/ grey literature to be searched include various targeted repositories, websites and databases. These include global organisations (e.g., the WHO,

For peer review only

United Nation Children's Fund [UNICEF], United Nations Office for Disaster Risk Reduction [UNDRR], United Nations International Strategy for Disaster Reduction [UNISDR], International Federation of Red Cross [IFRC], International Committee of the Red Cross [ICRC]), regional WHO offices (i.e., Southeast Asian, African, Western Pacific, Pan American, European and Eastern Mediterranean) and the European Centre for Disaster Medicine (CEMEC). Societies and organisations include the World Association for Disaster and Emergency Medicine (WADEM), Médecins Sans Frontières (MSF) and ReliefWeb. National websites include the United States Centers for Disease Control and Prevention (CDC) and Federal Emergency Management Agency (FEMA), the Robert Koch Institute (RKI), Public Health England. Lastly, Evidence Aid will be included as an evidence repository.

## Selection of studies

All search hits will be imported into Rayyan V0.1.0 software (Rayyan Systems Inc., MA, USA) (14) for screening, checking of duplicates and selection of final documents to be included. To support consistent abstract and title screening and refine eligibility, senior authors (RE, HG and MM) together with the title and abstract screeners (MP and MYC), will (as an initial step) independently and in duplicate screen 100 articles, followed by discussion. The following proposed screening approach is adapted from the Cochrane Rapid Reviews Methods Group guidance for systematic reviews to balance rigour and speed consistent with rapid reviews (15,16). Twenty percent of titles and abstracts will be screened by two reviewers (MP and MYC), independently, in duplicate and with conflict resolution, to remove obviously irrelevant reports. After this, one reviewer (MP) will screen the remaining titles and abstracts while the second reviewer (MYC) will verify excluded titles and abstracts and resolve conflicts (15). If required, a third senior reviewer (HG or RE) will resolve any disagreements The full texts of selected citations will subsequently be assessed in detail against the eligibility criteria by the first reviewer, while the second reviewer will verify all excluded full texts (15). Reasons for exclusion of sources of evidence at full text that do not meet the inclusion criteria will be recorded. This information will be reported and added to a table of excluded studies in the scoping review. Any disagreements that arise between the reviewers at each stage of the selection process will be resolved through discussion, including with an additional senior reviewer (HG or RE) if needed. If study eligibility is unclear owing to missing data, further information will be requested from study authors. The results of the search and the source inclusion process will be reported in full in the final scoping review and presented in a PRISMA-ScR flow diagram (12).

## Data extraction and management

Due to the rapid design and potential large pool of included studies we will use a dynamic approach to data extraction and management. For an included study yield of ≤25, data extraction will be done by one reviewer (MP), while a second reviewer (KB or MYC) will check for completeness and accuracy (15). For yields between >25 but ≤75, two or more extractors will be used (e.g., MP/KB/MYC/CJ/QL/RE), while an additional reviewer will check for

For peer review only

correctness and accuracy (17). In the case of more than 75 included sources, we will consider a prioritisation process whereby we rank or stratify studies based on design and relevancy to the scoping review. Prioritised studies will then be included for data extraction until the review team, together with WHO, agrees that data saturation has been achieved. The reviewers will discuss the nature of the information that will be extracted before commencing the process to facilitate coherence. Any uncertainties before and during the extraction process will be discussed with team members to make a final decision.

The data extracted will include author name(s), publication year, publication country and World Bank classification, source classification as primary/ secondary/ multi-method, publication type, study design, aim/ purpose, sample/ facility description, method/ tool for data collection, modifications to the data tool (if any), level (community, national, etc.), type of emergency, operational readiness definition, preparedness definition, key actors, challenges/ recommendations, lessons learnt, and other relevant information/ conclusions. In addition, data regarding readiness will be extracted according to the WHO's operational readiness components – these include:

- Leadership, governance and coordination,
- Country risk profile,
- Operational planning and coordination,
- Contingency finance,
- Health facility capacity and service delivery,
- Health workforce/ human resources,
- Early warning or surveillance and health information systems,
- Community resilience and risk communications,
- Logistics or supply chain for access to essential medicines,
- WHO readiness,
- Partner readiness.

Framework details and any associated actions will be recorded. Finally, information regarding relevant models will be extracted, including URL links to figures/ diagrams.

A draft extraction form is provided as Appendix II. The draft form will be pilot-tested independently by two reviewers using a sample of two to three potential included full-text articles/ evidence sources (17). Based on feedback from the two reviewers, the form may be modified and revised as necessary during the process of extracting data from each included evidence source (17). Necessary modifications will first be discussed within the review team for consensus, and any changes implemented will be reported in the final scoping review. Authors will be contacted where possible to clarify or obtain additional information.

For peer review only

## Methodological appraisal

Included peer-reviewed literature will be evaluated for quality based on appropriate pre-existing methodological quality checklists.

## Data analysis and presentation

Data will be synthesised in line with the core objectives of the rapid scoping review.

The included documents will be analysed using qualitative thematic analysis through an deductive synthesis approach (18–20). We are proposing to use ATLAS.ti V8 (Scientific Software Development GmbH) (https://atlasti.com/) to conduct thematic data analysis as well as store, organise, and retrieve data. Data analysis will be carried out by the project group researchers, who have vast knowledge and experience in undertaking reviews, including scoping reviews, that have used qualitative thematic analysis.

Findings will be deductively coded into a conceptual model that is taken from the WHO Country Readiness for Health Checklist to define and identify the critical elements of 'operational readiness' for public health emergencies, including COVID-19, and identify lessons learnt from addressing it. We will also identify if there are additional consistent themes emerging from the analysis that are not currently included in the WHO Checklist, as potential additional items.

The analysis will start by evaluating documented text line-by-line, allocating text a descriptive label and code. The same will be done for the other focused questions on understanding the similarities and differences between operational readiness and preparedness and identifying critical elements. The researchers will remain close to the data from the primary sources when defining and understanding the meaning structure of these concepts and phenomena. Since the conceptual understanding of 'operational readiness' and 'preparedness' will be initially explored, described, and theorised and may vary across sources, we will initially use broad, higher order codes (which may form main themes) developed deductively from the framework to organise the data. Once all data have been initially coded and collated, all the potentially relevant coded data extracts will be sorted and collated into themes and sub-themes (including a 'miscellaneous' theme for codes that do not clearly fit into existing themes (20). Senior reviewers (RE, HG and QL) will debrief the researchers primarily responsible for the thematic analysis, and the review team will meet regularly to discuss codes and themes, including potential merging or further break-down of themes (depending on whether there are enough data to support a theme, or the data are deemed too diverse). The themes will represent the synthesis and interpretation that go beyond the primary sources as well as deliver new insights and knowledge, which will translate into an operational framework for readiness and important lessons learnt.

A numerical description of the extent and nature of included evidence sources will be presented using tables and charts, accompanied by narrative summaries to describe how the results relate to the review's objectives.

## Reporting and dissemination

The Stellenbosch University (SU) review team will work with the WHO commissioning group and draw on the expertise of expert advisors to the review team to produce the following outputs. Weekly internal and SU-WHO meetings have been conducted to provide input into the development of this research protocol and will continue to aid understanding of emerging insights and findings that can inform work tasks relevant to the technical product development. Interim findings from the rapid scoping review will be presented to the WHO. Following feedback, an updated interim report incorporating feedback from the WHO and expert advisory team will be presented. The final report of the full rapid scoping review will be delivered by December 2021, along with a PowerPoint presentation to the WHO commissioning group of findings with talking points. In consultation with the WHO, findings will be disseminated further as appropriate (e.g., through professional bodies, conferences, and research papers). By defining evidence related to critical readiness components and actions, this review will reveal new insights, knowledge and lessons learnt that will translate into an operational framework for readiness actions.

## Ethics and Patient and Public Involvement

No ethical approval is needed for this rapid scoping review, given that included sources will comprise of published and publicly available information.

## Contributorship statement

**RE** is the principle investigator for the project and conceptualised and guided the writing process, **JN** is the project coordinator, drafted the background and objectives of the proposal and **HG, QL, MM, CJ, MO** and **KB** conceptualised and wrote up the methodology section of the proposal. In addition to methodology, **KB** compiled and edited the overall proposal. **JN** is the corresponding author.

## Acknowledgements

The authors would like to acknowledge Professor Tarryn Young for technical guidance and insights and the World Health Organisation which commissioned the review to inform an Operational Readiness Framework for the Country Readiness Strengthening Department in the World Health Emergencies Program in WHO (Reference #: 2021/1145765; Unit: MST; Cluster: QNF/SCI).

For peer review only

## Funding

This work is supported by the World Health Organization [Reference: APW/RR/Readiness/2021/1145765].

## Conflicts of interest

There is no conflict of interest in this project.

For peer review only

## References

1. Savoia E, Lin L, Viswanath K. Communications in public health emergency preparedness: A systematic review of the literature [Internet]. Vol. 11, Biosecurity and Bioterrorism. Mary Ann Liebert, Inc.; 2013 [cited 2021 Oct 4]. p. 170–84. Available from: /pmc/articles/PMC3778998/

2. Tang R, Fitzgerald G, Hou XY, Wu YP. Building an evaluation instrument for china's hospital emergency preparedness: A systematic review of preparedness instruments [Internet]. Vol. 8, Disaster Medicine and Public Health Preparedness. Lippincott Williams and Wilkins; 2014 [cited 2021 Oct 4]. p. 101–9. Available from: https://pubmed.ncbi.nlm.nih.gov/24622400/

3. Ejeta LT, Ardalan A, Paton D. Application of behavioral theories to disaster and emergency health preparedness: A systematic review. PLoS Curr [Internet]. 2015 Jul 1 [cited 2021 Oct 4];7(DISASTERS). Available from: https://pubmed.ncbi.nlm.nih.gov/26203400/

4. WHO Emergencies Preparedness Team. Emergency response framework [Internet]. 2nd ed. World Health Organization, editor. Geneva; 2017. Available from: https://www.who.int/publications/i/item/9789241512299

5. Lo STT, Chan EYY, Chan GKW, Murray V, Abrahams J, Ardalan A, et al. Health Emergency and Disaster Risk Management (Health-EDRM): Developing the Research Field within the Sendai Framework Paradigm. Int J Disaster Risk Sci [Internet]. 2017 Jun 1 [cited 2021 Oct 4];8(2):145–9. Available from: www.ijdrs.comwww.springer.com/13753

6. WHO Emergencies Preparedness Team. A strategic framework for emergency preparedness [Internet]. World Health Organization, editor. Geneva; 2016. Available from: https://www.who.int/publications/i/item/a-strategic-framework-for-emergency-preparedness

7. Haldane V, De Foo C, Abdalla SM, Jung AS, Tan M, Wu S, et al. Health systems resilience in managing the COVID-19 pandemic: lessons from 28 countries [Internet]. Vol. 27, Nature Medicine. Nature Research; 2021 [cited 2021 Oct 4]. p. 964–80. Available from: https://pubmed.ncbi.nlm.nih.gov/34002090/

8. Khan Y, O'Sullivan T, Brown A, Tracey S, Gibson J, Généreux M, et al. Public health emergency preparedness: A framework to promote resilience. BMC Public Health [Internet]. 2018 Dec 5 [cited 2021 Oct 4];18(1):1344. Available from: https://bmcpublichealth.biomedcentral.com/articles/10.1186/s12889-018-6250-7

9. Peters M, Godfrey C, McInerey P, Munn Z, Tricco A, Khalil H. Chapter 11: Scoping Reviews (2020 version). In: Aromataris E, Munn Z, editors. JBI Manual for Evidence Synthesis [Internet]. JBI; 2020. Available from: https://synthesismanual.jbi.global

10. Nelson C, Lurie N, Wasserman J, Zakowski S. Conceptualizing and defining public health emergency preparedness [Internet]. Vol. 97 Suppl 1, American journal of public

For peer review only

health. American Public Health Association; 2007 [cited 2021 Oct 5]. p. S9–11. Available from: /pmc/articles/PMC1854988/

11. World Health Organization. Chapter 11: Public health emergencies. In: Advancing the right to health: the vital role of law [Internet]. Geneva: World Health Organization; 2017. Available from: https://apps.who.int/iris/handle/10665/252815

12. Tricco AC, Lillie E, Zarin W, O'Brien KK, Colquhoun H. PRISMA Extension for Scoping Reviews (PRISMA-Scr): Checklist and Explanation. Ann Intern Med. 2018;467–73.

13. Rethlefsen ML, Kirtley S, Waffenschmidt S, Ayala AP, Moher D, Page MJ, et al. PRISMA-S: an extension to the PRISMA Statement for Reporting Literature Searches in Systematic Reviews. Syst Rev [Internet]. 2021 Jan 26 [cited 2021 Oct 4];10(1):39. Available from: https://doi.org/10.1186/s13643-020-01542-z

14. Ouzzani M, Hammady H, Fedorowicz Z, Elmagarmid A. Rayyan -a web and mobile app for systematic reviews. Syst Rev [Internet]. 2016 [cited 2021 Oct 4];5(1):210. Available from: http://systematicreviewsjournal.biomedcentral.com/articles/10.1186/s13643-016-0384-4

15. Garritty C, Gartlehner G, Nussbaumer-Streit B, King VJ, Hamel C, Kamel C, et al. Cochrane Rapid Reviews Methods Group offers evidence-informed guidance to conduct rapid reviews. J Clin Epidemiol [Internet]. 2021 Feb 1 [cited 2021 Oct 4];130:13–22. Available from: https://doi.org/10.1016/j.jclinepi.2020.10.007

16. Tricco AC, Garritty CM, Boulos L, Lockwood C, Wilson M, McGowan J, et al. Rapid review methods more challenging during COVID-19: commentary with a focus on 8 knowledge synthesis steps [Internet]. Vol. 126, Journal of Clinical Epidemiology. Elsevier Inc.; 2020 [cited 2021 Oct 6]. p. 177–83. Available from: http://www.jclinepi.com/article/S0895435620306168/fulltext

17. Peters MDJ, Marnie C, Tricco AC, Pollock D, Munn Z, Alexander L, et al. Updated methodological guidance for the conduct of scoping reviews. JBI Evid Synth [Internet]. 2020 Oct 1 [cited 2021 Oct 6];18(10):2119–26. Available from: https://journals.lww.com/10.11124/JBIES-20-00167

18. Thomas J, Harden A. Methods for the thematic synthesis of qualitative research in systematic reviews. BMC Med Res Methodol [Internet]. 2008 Dec 10 [cited 2021 Oct 5];8(1):45. Available from: https://bmcmedresmethodol.biomedcentral.com/articles/10.1186/1471-2288-8-45

19. Levac D, Colquhoun H, O'Brien KK. Scoping studies: advancing the methodology. Implement Sci. 2010 Sep;5:69.

For peer review only

# Draft search strategies

| Database | Query | | Records retrieved |
|---|---|---|---|
| **MEDLINE** | **Ovid MEDLINE(R) and In-Process, In-Data-Review & Other Non-Indexed Citations <1946 to September 27, 2021>** | | |
| | 1 | Disease Outbreaks/ | 85271 |
| | 2 | epidemics/ or pandemics/ | 77571 |
| | 3 | (catastrophe* or disaster* or drought* or earthquake* or evacuation* or famine* or flood or floods or hurricane or cyclone* or landslide* or landslide* or tsunami* or tidal wave* or volcano*).tw. | 95527 |
| | 4 | Natural Disasters/ | 348 |
| | 5 | Public health emergency.mp. | 2882 |
| | 6 | COVID-19/ | 105511 |
| | 7 | Covid*.tw. | 132306 |
| | 8 | Hemorrhagic Fever, Ebola/ | 6009 |
| | 9 | Ebola virus.mp. or Ebolavirus/ | 6362 |
| | 10 | SARS Virus/ or SARS-CoV-2/ or SARS.mp. | 113897 |
| | 11 | yellow fever.tw. or Yellow Fever/ | 7064 |
| | 12 | Influenza, Human/ | 52855 |
| | 13 | Communicable Diseases, Emerging/ | 6305 |
| | 14 | emerging virus*.mp. | 1360 |
| | 15 | 1 or 2 or 3 or 4 or 5 or 6 or 7 or 8 or 9 or 10 or 11 or 12 or 13 or 14 | 407332 |
| | 16 | Disaster Planning/ or preparedness.tw. | 27006 |
| | 17 | readiness.tw. | 17895 |
| | 18 | risk assessment.tw. or Risk Assessment/ | 322294 |
| | 19 | Disaster Medicine/ or *"Emergency Medical Services"/ | 33078 |
| | 20 | public health response.mp. | 1632 |
| | 21 | emergency planning.mp. | 501 |
| | 22 | disaster management.mp. | 1370 |
| | 23 | operational readiness.mp. | 169 |
| | 24 | Leadership/ | 43759 |
| | 25 | governance.tw. | 14287 |
| | 26 | Incident Management System.mp. | 111 |
| | 27 | Emergency Operations Centre.mp. | 27 |
| | 28 | multi-sector coordination.mp. | 3 |
| | 29 | Country Risk Profile.mp. | 0 |
| | 30 | Operational planning.mp. | 174 |
| | 31 | ("emergency response plan*" or "contingency plan*").mp. [mp=title, abstract, original title, name of substance word, subject heading word, floating sub-heading word, keyword heading word, organism supplementary concept word, protocol supplementary concept word, rare disease supplementary concept word, unique identifier, synonyms] | 1177 |
| | 32 | (logistics and supply chain).mp. [mp=title, abstract, original title, name of substance word, subject heading word, floating sub-heading word, keyword heading word, organism supplementary | 267 |

For peer review only



For peer review only - http://bmjopen.bmj.com/site/about/guidelines.xhtml

| | | | |
|---|---|---|---|
| | | concept word, protocol supplementary concept word, rare disease supplementary concept word, unique identifier, synonyms] | |
| | 33 | Contingency finance.mp. | 0 |
| | 34 | Drugs, Essential/ or essential medicine*.mp. | 1977 |
| | 35 | logistic supply.mp. | 7 |
| | 36 | early warning.mp. | 7531 |
| | 37 | Public Health Surveillance/ | 4719 |
| | 38 | community resilience.mp. | 561 |
| | 39 | 16 or 17 or 18 or 19 or 20 or 21 or 22 or 23 or 24 or 25 or 26 or 27 or 28 or 29 or 30 or 31 or 32 or 33 or 34 or 35 or 36 or 37 or 38 | 466764 |
| | 40 | 15 and 39 | 29015 |
| | 41 | health system*.mp. | 61652 |
| | 42 | health services.tw. or Health Services/ | 95945 |
| | 43 | Public Health/ | 87343 |
| | 44 | health facilities.mp. or Health Facilities/ | 29065 |
| | 45 | health personnel.mp. or exp Health Personnel/ | 625414 |
| | 46 | 41 or 42 or 43 or 44 or 45 | 857447 |
| | 47 | 40 and 46 | 5815 |
| | 48 | limit 47 to yr="2010 -Current" | 4275 |
| **Embase** | **Embase <1996 to 2021 Week 38>** | | |
| | 1 | Disease Outbreak*.tw. | 7421 |
| | 2 | epidemic/ or pandemic/ | 187037 |
| | 3 | (catastrophe* or disaster* or drought* or earthquake* or evacuation* or famine* or flood or floods or hurricane or cyclone* or landslide* or landslide* or tsunami* or tidal wave* or volcano*).tw. | 96165 |
| | 4 | Public health emergency.mp. | 3299 |
| | 5 | natural disaster/ | 3548 |
| | 6 | Covid*.tw. | 157374 |
| | 7 | coronavirus disease 2019/ | 148801 |
| | 8 | Ebola hemorrhagic fever/ | 6883 |
| | 9 | Ebolavirus/ | 3761 |
| | 10 | SARS.mp. or severe acute respiratory syndrome/ | 80752 |
| | 11 | yellow fever/ | 3932 |
| | 12 | *influenza/ | 25733 |
| | 13 | Communicable Diseases, Emerging/ | 12560 |
| | 14 | 1 or 2 or 3 or 4 or 5 or 6 or 7 or 8 or 9 or 10 or 11 or 12 or 13 | 442828 |
| | 15 | Disaster Planning/ or preparedness.tw. | 26939 |
| | 16 | readiness.tw. | 21708 |
| | 17 | risk assessment.tw. or Risk Assessment/ | 630413 |
| | 18 | public health response.mp. | 1779 |
| | 19 | emergency planning.mp. | 656 |
| | 20 | disaster management.mp. | 1674 |
| | 21 | operational readiness.mp. | 202 |
| | 22 | Leadership/ | 70482 |
| | 23 | governance.tw. | 18071 |
| | 24 | Incident Management System.mp. | 154 |
| | 25 | Emergency Operations Centre.mp. | 36 |
| | 26 | multi-sector coordination.mp. | 6 |
| | 27 | Country Risk Profile.mp. | 0 |

For peer review only

| | | | |
|---|---|---|---|
| | 28 | Operational planning.mp. | 204 |
| | 29 | ("emergency response plan*" or "contingency plan*").mp. | 1478 |
| | 30 | (logistics and supply chain).mp. | 417 |
| | 31 | Contingency finance.mp. | 0 |
| | 32 | logistic supply.mp. | 19 |
| | 33 | early warning.mp. | 9804 |
| | 34 | Public Health Surveillance.tw. | 2351 |
| | 35 | community resilience.mp. | 576 |
| | 36 | health system*.mp. | 81325 |
| | 37 | health services.tw. or Health Service/ | 196744 |
| | 38 | Public Health/ | 177806 |
| | 39 | emergency health service/ | 91713 |
| | 40 | disaster medicine/ | 1419 |
| | 41 | essential drug/ | 1412 |
| | 42 | essential medicine*.tw. | 2275 |
| | 43 | 15 or 16 or 17 or 18 or 19 or 20 or 21 or 22 or 23 or 24 or 25 or 26 or 27 or 28 or 29 or 30 or 31 or 32 or 33 or 34 or 35 or 36 or 37 or 38 or 39 or 40 or 41 or 42 | 860867 |
| | 44 | 14 and 43 | 42998 |
| | 45 | health care facility/ | 62270 |
| | 46 | health personnel.mp. or health care personnel/ | 247442 |
| | 47 | 28 or 29 or 30 or 46 or 47 | 688599 |
| | 48 | 44 and 47 | 9874 |
| | 49 | limit 48 to yr="2010 -Current" | 7852 |
| | 50 | limit 49 to exclude medline journals | 1076 |
| **Web of Science** | **Science Citation index expanded and Social Sciences citation index (Web of Science)** | | |
| | 1 | epidemic* or pandemic* or "natural disaster*" or earthquake* or evacuation* or famine* or flood or floods or hurricane or cyclone* or landslide* or landslide* or tsunami* or Covid-19 or Ebola or "yellow fever" or "human influenza" or "emerging diseases" (Topic) | 617867 |
| | 2 | preparedness or readiness or "risk assessment" or "Emergency Medical Services" or "public health response" or "emergency planning" or "disaster management" (Topic) or Leadership or governance or "Incident Management System" or "Emergency Operations " (Topic) or logistics or "supply chain" or "essential drugs" or "early warning" (Topic) | 813269 |
| | 3 | (#2) AND #1 | 38333 |
| | 4 | (#2) AND #1 and 2022 or 2021 or 2020 or 2019 or 2018 or 2017 or 2016 or 2015 or 2014 or 2013 or 2012 or 2011 or 2010 (Publication Years) | 33618 |
| | 5 | ((#2) AND #1) AND TS=("health system*" or "health services" or "health facilities" or "health personnel") | 1624 |
| | 6 | ((#2) AND #1) AND TS=("health system*" or "health services" or "health facilities" or "health  personnel") and 2022 or 2021 or 2020 or 2019 or 2018 or 2017 or 2016 or 2015 or 2014 or 2013 or 2012 or 2011 or 2010 (Publication Years) | 1476 |



For peer review only - http://bmjopen.bmj.com/site/about/guidelines.xhtml

| Author(s) | Publication year | Country | World Bank classification | Primary/ Secondary/ Multi-method | Publication type: Journal Article/ Opinion/ Guideline/ Organisational Framework/ Systematic Review/ Scoping review/ Narrative review) | Study design (where relevant) | Aim/ purpose | Sample/ Facility Description | Method/ tool for data collection | Modifications to data tool | Level (community, national, etc.) | Type of emergency | Operational readiness definition | Preparedness definition | Key actors |
|---|---|---|---|---|---|---|---|---|---|---|---|---|---|---|---|
| **Ogoina et al.** | 2021 | Nigeria | Middle-income | Primary | Journal article | Descriptive cross-sectional | To determined the level of readiness of hospitals in Nigeria to respond to COVID-19 | 20 secondary and tertiary healthcare facilities designated COVID facilities | WHO COVID-19 hospital readiness checklist | **Staff wellfare** (Hospital has secure accommodation for staff involved in the care of COVID-19; Hospital has plans to cater for feeding of staff dedicated for COVID-19 care; Hospital has plans for any prophylaxis for staff caring for COVID-19 patients; There is a special remuneration for staff involved in COVID-19 patient care; There is a health/life insurance for staff involved in COVID-19 patient care and availability of critical items **Availability of critical items** (e.g. PPE) | National | COVID-19 | Not mentioned | Not mentioned | Not mentioned |
| **Burkle et al.** | 2011 | USA | High-income | Multi-method | Journal article | Recommendation development expert group | This article addresses the role of community preparedness in establishing and empowering resilience and in mitigating the transmission of disease. In doing so, it ensures greater opportunities for critical care to reach a larger population of children. | 17-member Steering Committee with guidance from members of the American Academy of Pediatrics;<br>Pediatric Emergency Mass Critical Care Task Force (36 experts from diverse public health, medical, and<br>disaster response fields) | Recommendation development by expert groups informed by literature reviews of MEDLINE and Ovid databases | N/A | Community | Primary focus on pandemics although the preparedness aspects apply to all public health emergencies (PHEs). | Not mentioned | Not mentioned | The recommendations are made for pediatric leadership (those who represent, care for, and advocate for children) |

| Author(s) | Leardership or governance and coordination (subcomponent/s) | Actions | Timing (when) | Country risk profile (any description) | Actions | Timing (when) | Operational planning and coordination (subcomponent/s) | Actions | Timing (when) | Contingency finance (description) | Actions | Timing (when) |
|---|---|---|---|---|---|---|---|---|---|---|---|---|
| **Ogoina et al.** | | | | | | | | | | | | |
| **Burkle et al.** | Not mentioned | N/A | N/A | Not mentioned | N/A | N/A | Not mentioned | N/A | N/A | Not mentioned | N/A | N/A |

For peer review only

| Health facility capacity and service delivery (subcomponent/s) | Actions | Timing (when) | Health workforce/human resources (Main findings) | Actions | Timing (when) | Early warning surveillance or health information systems (subcomponent/s) | Actions | Timing (when) | Community resilience and risk communications (subcomponent/s) | Actions | Timing (when) | Logistics or supply chain for access to essential medicines | Actions | Timing (when) | WHO readiness (description) | Actions | Timing (when) | Partner readiness (description) | Actions | Timing (when) | Challenges/ Recommendations | Lessons learnt | Other relevant information/ Conclusion |
|---|---|---|---|---|---|---|---|---|---|---|---|---|---|---|---|---|---|---|---|---|---|---|---|
| **Infection prevention and control (IPC):** Most hospitals had completed indicators related to IPC, including training of healthcare workers (HCW) on IPC (75%), provision of IPC resources (80%) and compliance with isolation (80%), and droplet/contact precautions (80%) by HCW (Table 4). Most hospitals (80%) also have a team of HCW designated to care exclusively for suspected and confirmed cases ofCOVID 19. However, only 50% of hospitals had certified that HCW are applying standard precautions, while only 40% reported having adequate personal protective equipment (PPE) for HCW.<br><br>**Continuity of essential health services, and surveillance:** Table 7 shows that most hospitals (70%) maintained essential hospital services while only 30% have listed all hospital services in order of priority. Regarding surveillance, 80% of hospitals ensured COVID-19 testing is done in line with national criteria but only eight hospitals (40%) each have appointed a hospital epidemiologist to coordinate surveillance and identified the information that needs to be collected and defined the objectives for its use.<br><br>**Communication:** Twelve hospitals (70.6%) ensured decisions related to management of COVID-19 are communicated to all relevant staff and stakeholders, 55% have briefed staff on their roles and responsibilities regarding care of COVID-19 and 60% have appointed a public information spokesperson to coordinate communication.<br><br>**Logistic, supplies and pharmaceuticals:** About 40% of hospitals had an updated inventory of all equipment, supplies, and pharmaceuticals, and reordering mechanism. Only seven hospitals (35%) had estimated the consumption of essential equipment, supplies, and pharmaceuticals and 25% had established contingency agreements with vendors to ensure the procurement and prompt delivery of equipment, supplies, and other resources. | Survey of hospital readiness | After first case was identified | **COVID-19-related specialist human resources:** The total number of full-time specialist human resource for COVID-19 response ranged from 20 for infectious diseases (ID) physicians to 111 for public health physicians (Table 1). Majority of hospitals (17 of 20) had at least one anaesthesiologist on full term employment, while only 11 hospitals had ID physicians on full term employment. About 90% of ID physicians had been trained on COVID-19 intervention, while about 53.2% of public health physicians and 28.8% of anaesthesiologists had prior training for COVID-19 intervention. | Survey of hospital readiness | After first case was identified | | | | | | | **Laboratory services and essential support services:** Sixteen (80%) hospitals had ensured the continuous availability of basic laboratory testing, but only seven hospitals (40%%) had established a laboratory referral pathway for the identification, confirmation, and monitoring of COVID-19 (Table 10). About 70% of hospitals had trained staff on specimen handling, packaging, and transportation procedures for specimen referrals, 50% of hospitals had established a biosafety level 2 or higher laboratory for diagnosis of infectious diseases, and 40% had the capability to test for COVID-19. | Survey of hospital readiness | After first case was identified | | | | | | | | | Most hospitals in Nigeria were not adequately prepared to respond to the COVID-19 outbreak. Current efforts to strengthen hospital preparedness should prioritize challenges related to surge capacity, critical care for COVID-19 patients, and staff welfare and protection. |
| Not mentioned | N/A | N/A | Work with community planners to identify the logistic support necessary for establishing and operating **alternate care facilities**, and identify and create **protocol-driven patient management objectives** based on assumptions about the types of patients that would be managed in such facilities. | Recommendation | | Advocate for 9-1-1 **telephone triage with pre-established criteria** and **protocols for the proper use and safety of EMS and EMS-sanctioned transportation** during pandemics. | Recommendation | | Actively promote programs to ensure, before and during a crisis, an **informed citizenry** and the **education** of children and families. Promote programs for an informed citizenry and encourage the inclusion of evidence that community and faith-based organizations have both received and learned (knowledge transferred) the **recommended preparedness/ emergency information** as cited in the Public Health Preparedness Capabilities: National Standards for State and Local Planning, particularly as recommended in the Community Preparedness and Medical Surge sections.<br><br>Advocate for a **community level of preparedness** that leads to **empowered** self-awareness, **knowledge** of the information that best prepares the public to provide basic lifesaving information and self-care, and builds physical and mental health **resilience**.<br><br>Advocate for the establishment of permanent national and state level **call systems** and for disease- and child-specific **health lines** as crucial adjuncts during public health emergencies.<br><br>Advocate for creative operational concepts that provide **guidance and protocols sensitive to the needs of the pediatric population**. This includes means to optimize critical care opportunities at hospitals and critical care centers, case definition-driven triage management protocols that provide input from pediatricians and society alike, and representation of pediatric leadership on community level decision-making bodies, such | Recommendation | | Not mentioned | N/A | N/A | Not mentioned | N/A | N/A | Not mentioned | N/A | N/A | The Pediatric Emergency Mass Critical Care Task Force recommends the following actions by pediatric leadership: active promotion of programs to ensure an informed citizenry; education of children and families in Centers for Disease Control and Prevention community mitigation strategies; emphasis on community-level preparedness empowering the public to provide self care; use of 9-1-1 telephone triage with pre-established protocols and in coordination with emergency medical services; and advocacy for healthcare coalitions and other creative operational concepts that provide guidance and protocols for care of the pediatric population. | | |

For peer review only

| Operational Readiness Component | Sub-Component |
|---|---|
| Leadership/Governance (HS BB#6) and Coordination HEDRM POLICIES, STRATEGIES AND LEGISLATION | Country has a legal framework for emergency preparedness, response and recovery actions |
| | HS Gov readiness add here |
| | Coordination of Incident Management Systems. Coordinate multiple IMSs, each sector needs SOPs to activate IMS and interoperate with others with capacity for standard IMS and avoid duplication |
| | Emergency Operations Centre (or equivalent) is well functioning and adequately staffed |

| | |
|---|---|
| | |
| | The health sector is well-integrated in multi-sectorial coordination |
| Country Risk Profile | Country has developed an updated country risk profile |
| | Country has developed a national emergency response plan for the health sector |
| | |

| | |
|---|---|
| **Operational Planning ERDRM PLANNING AND COORDINATION** | **Country has developed contingency plans based on risk profile/assessment and vulnerabilities** |
| | **Points of Entry** |

| | |
|---|---|
| | |
| | **Logistics and Supply Chain** |
| **Contingency Finance (HS BB#5) HEDRM FINANCIAL RESOURCES** | **Contingency Finance for Emergency Repsonse and Readiness, add HS Finance readiness here** |
| | |

Health Facility capacities to support emergency response are well-defined and mapped

Health facilities have contingency plans and business continuity plans for emergency response

Health Facility Capacity and Service Delivery (HS BB#1)

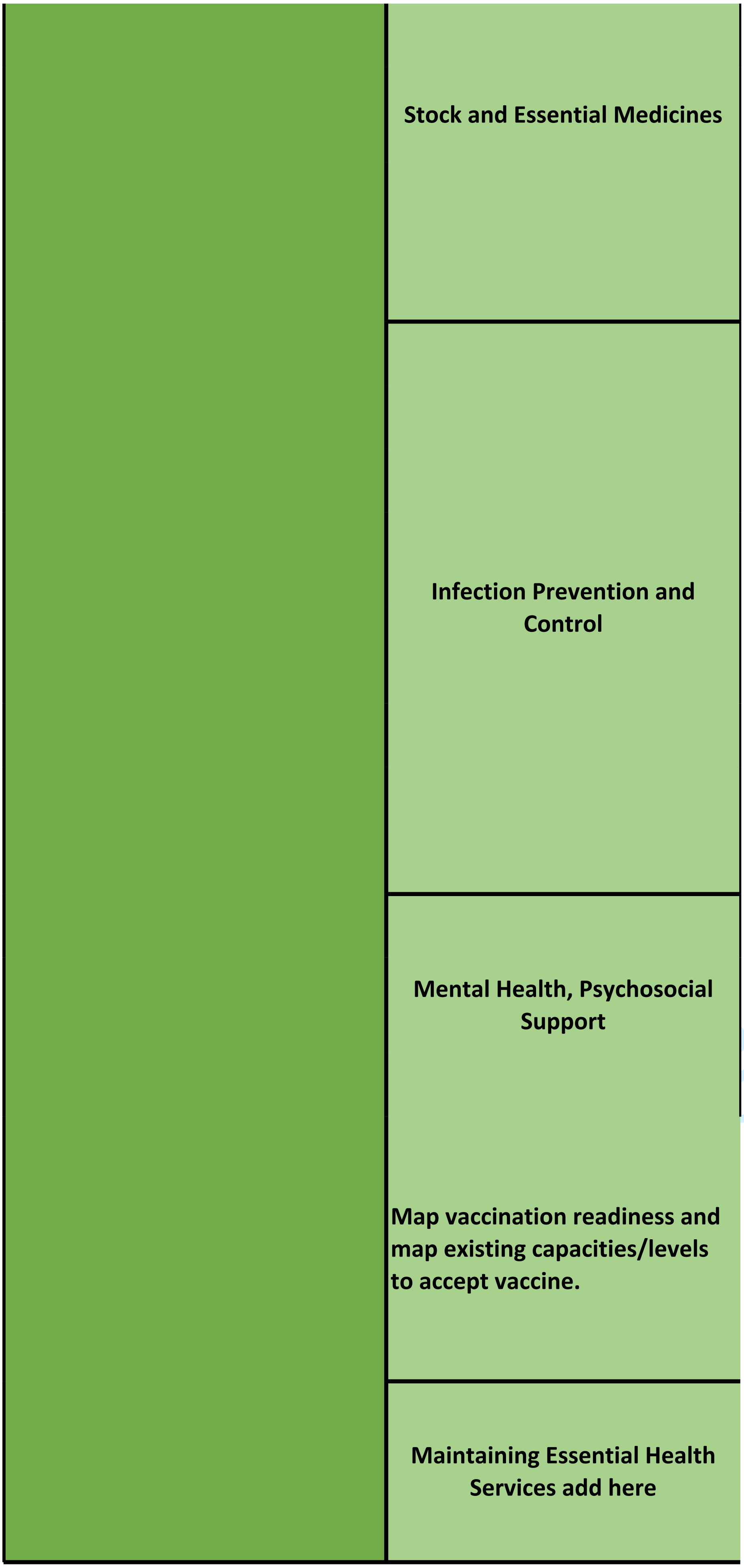
Stock and Essential Medicines
Infection Prevention and Control
Mental Health, Psychosocial Support
Map vaccination readiness and map existing capacities/levels to accept vaccine.
Maintaining Essential Health Services add here

| | |
|---|---|
| Health Workforce (HS BB#2) HEDRM HUMAN RESOURCES | Health Workforce Capacity in place, add here |
| | Qualified Surge Capacity for Emergency Response is available, add IHS here, Note: as part of Health Sector BCP |
| | External Surge Capacity mechanisms are well-defined in case Country requires external support to respond to emergency, add IHS here |
| | |

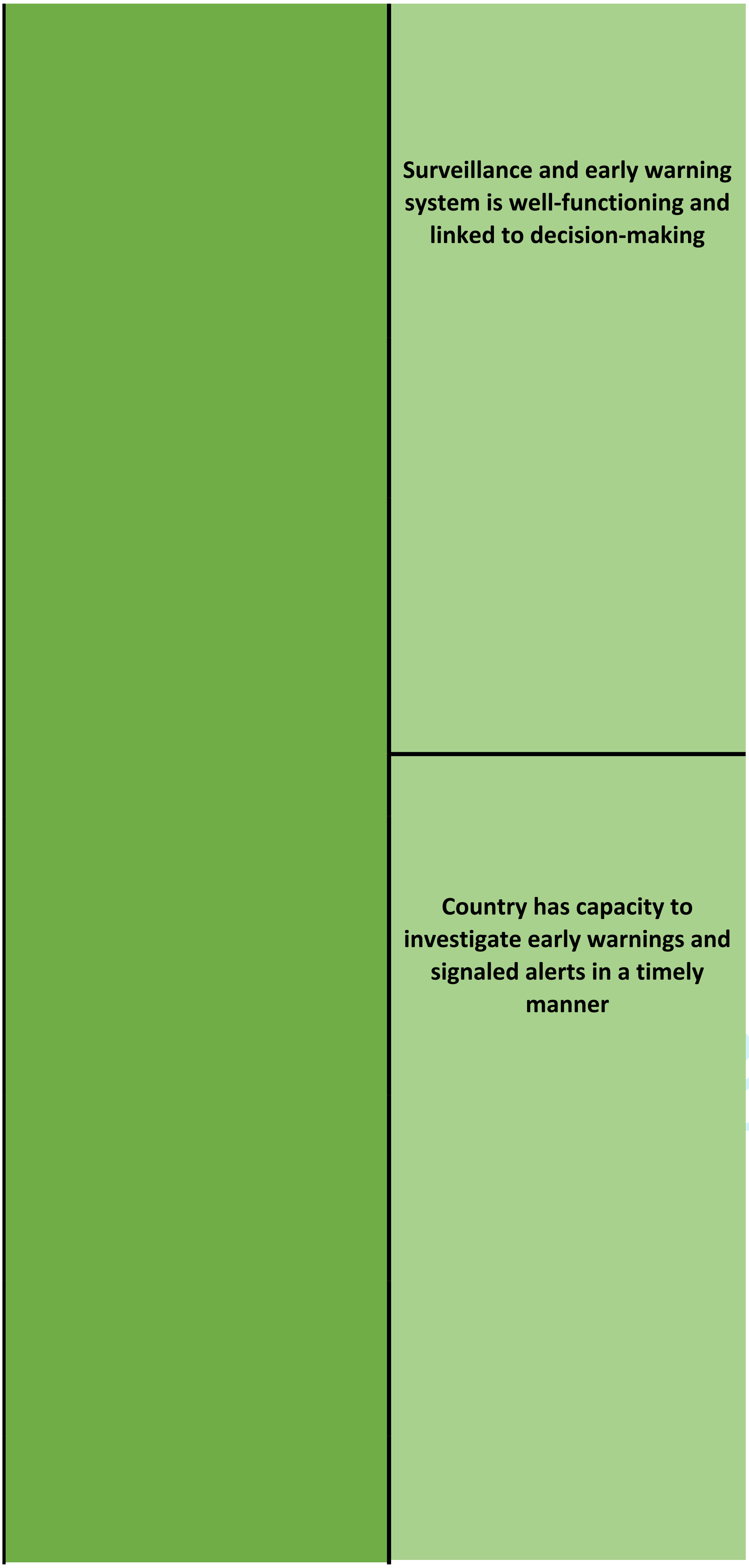


For peer review only - http://bmjopen.bmj.com/site/about/guidelines.xhtml

**Early Warnings/ Surveillance and Health Information Systems (HS BB#3). HEDRM INFORMATION AND KNOWLEDGE MANAGEMENT**

**Country has adequate lab capacity during emergency response**

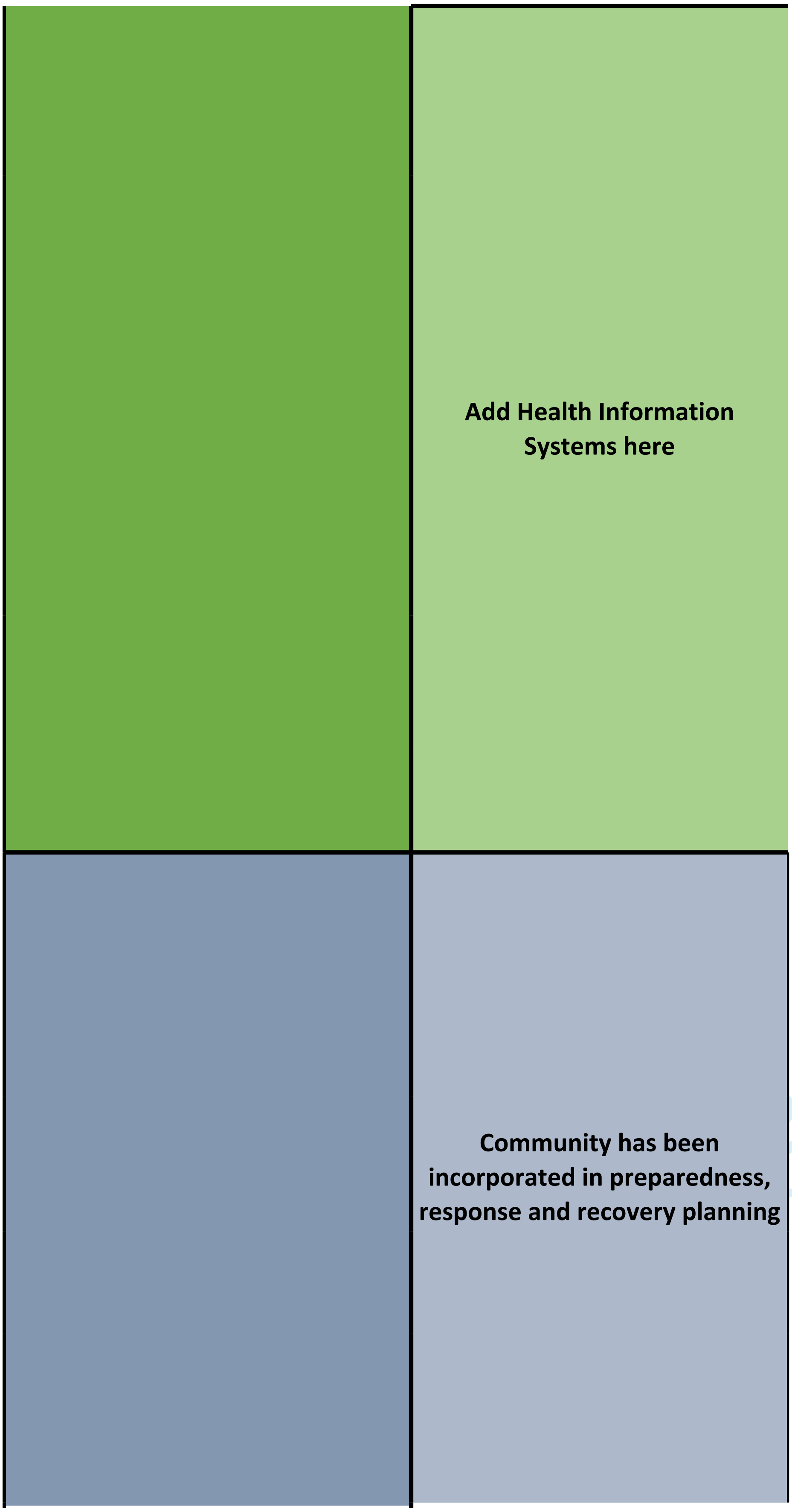
Add Health Information
Systems here
Community has been
incorporated in preparedness,
response and recovery planning

| | |
|---|---|
| Community Resilience and Risk Communications | |
| | **Risk Communication strategy for the health sector is ready during emergency** |
| | |

For peer review only - http://bmjopen.bmj.com/site/about/guidelines.xhtml

| | |
|---|---|
| | **Community Resilience: Readiness and Response** |
| **Logistics/ Supply Chain/Access to Essential Medicines (HS BB#4)** | **Country has capacity to pre-position medical supplies based on hazard, add HS, IHS here** |

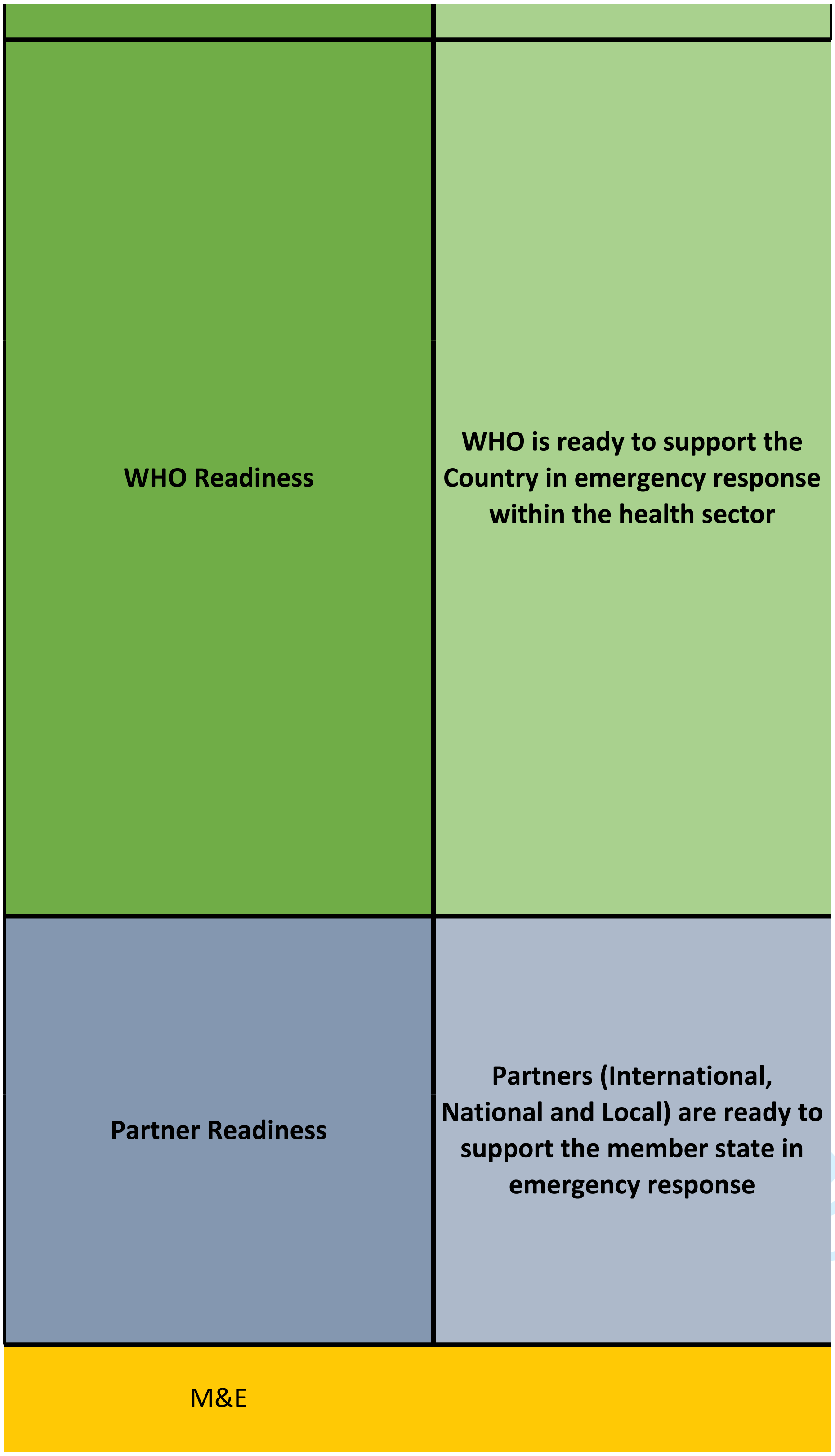
WHO Readiness
WHO is ready to support the Country in emergency response within the health sector
Partner Readiness
Partners (International, National and Local) are ready to support the member state in emergency response
M&E

For peer review only

| Tasks/Actions | Ogoina et al 2021 |
|---|---|
| **Country has an exclusive legal framework / legislation describing the legal mandate for emergency preparedness, response planning, and recovery actions with sector specific policies/provisions** | |
| | |
| **Health emergency response coordination mechanisms (such as health cluster, health sector coordinating body) established, well-functioning and include relevant sectors, public, private, civil society organizations and other relevant stakeholders and partners** | |
| Country has a designated health cluster/sector coordinator to lead health sector response coordination within health sector as well as amongst relevant partners | |
| Health sector/cluster response coordination mechasnism established and functional at different levels of health service delivery system including health services of Armed Forces at national, subnational and communities. | |
| Working mechanisms, mandates and expected contributions by all identified national and international stakeholders and partners are clearly outlined at national as well as subnational emergency response plans with MoUs or other collaborative agreements. These should be tested through simulation exercises at least once a year. | |
| Clear SoPs for cluster/sector coordination, health cluster/sector activation and deactivation during the emergency response under the overall national cluster/sector coordination should be reviewed and updated periodically and tested through the simulation exercises | |
| Country has well-qualified information management officer(s) within the health cluster (or equivalent coordinating health sector body) to support management of information and analysis amongst health sector actors. | |
| **Public health emergency operations centre(s) are well established and integrated/linked to any multisectoral EOCs** | |
| PHEOC has adequate staff that are well-trained in incident management and EOC critical functions | |
| PHEOC has adequate equipment including communications systems to respond during emergency response | |
| Countries ensure interoperability and functional linkage/integration between different EoCs within health sector and with EoCs at national emerency coordinating body and others sectors including WASH. | |
| **Excercising IMS in EOCs,** | |

| |
|---|
| Country has run the PHEOC framework checklist and addressed gaps to ensure continuity of EOC function |
| **The health sector is incorporated in the overall Country emergency/ coordination mechanism** |
| Health Sector represented in Emergency Prepardness and Response mechanisms not only at national but also at subnational and community levels along with all other key stakeholdrs in line with the national legislations or legal framework. |
| Country has a well defined coorindaiton mechansm and procedures in place to work with UN Country team including WHO and other international parters during the emergency preparedness, response and recovery phases to ensure international norms and standards are adapted into country context and technical and financal supports are mobilized quickly when needed. These mechanims should be tested during the national simulation exercises |
| **The country has an updated national risk assessment, which describes the main risks to the country (inclusive of exposure, hazard, vulnerability) as well as the seasonality of the identified risks** |
| Country has identified and described vulnerable populations and communities based on the identified risks |
| Countries with diversity and geographical variation of risks and decentralized governance and administrative system can conduct risk assessment at relevant subnational levels to identify risks specific and relevant to their areas |
| **Country has an updated emergency response plan for the health sector that is linked to the national emergency response (disaster response) plan** |
| Updated Emergency Preparedness and Response Plan contains as a minumum (1) Hazard and vulnerability analysis and risk mapping, (2) Mechanism for coordination, command and control, (3) Description of roles and responsibilities of different partners, (4) Pre-arrangement with partners (e.g. logistics support, medical supplies,(5) Provisions for implementation and operationalization of the plan (e.g. SoP). |
| The response plan and country capacities have been tested through simulation exercises within the last year and the plan has been updated |
| Country emergency response plan for health clearly links the national plan to any relevant subnational and local response plans |
| The health emergency response plan includes continuity of health services, roles and responsibilities of health workforce |
| **The country has regularly updated contingency plans based on identified risks and the risk profile** |

| |
|---|
| The Contingency plans should be hazard specific and scenario based targeting priority and high impact threats and risks identifed based on strategic risk assessment. |
| The contingency plans have been tested either through emergency response or simulation exercise in the past 2 years. |
| ToRs for each actors in the contigency plans are well specified and tested during the simulations and same for necessary MoUs or other collaborative agreements with partners |
| The contigency plans are revised based on results of simulation exercise or AARs and also reassessemment of country risks at least every two years. |
| Contingency plans link to the emergency response plan for health sector and national and subnational plans, and communities |
| All designated PoEs have public health emergency contingency plans developed and tested periodically and also review the plans to ensure the plans are fit-for-purpose for any ongoing outbreak/pandemic at country, regional and globally which may afffect the country |
| The contigency plans involve relevant sectors at PoEs with clearly defined duties and responsibilities of each sector and be part of PoE emergency plan |
| Test the procedures and means in place for communicating ill travellers between conveyances and PoEs, as well as between national health authorities (EWARS, PHEOC) |
| Ensure staff working at PoEs are aware of the appropriate actions to manage ill passenger (s) detected before boarding, onboard conveyances (airplane and ship), and on arrival at PoE |
| Develop or implement paper based and/or electronic system for storing, recording and disposing of records captured during entry. Such system should be applied fairly and lawfully while respecting passenger confidentiality |
| Establish and maintain two way communication mechanism between PoEs, national surveillance system and primary health care centers for communicating on development of signs and symptoms of infectious disease from recent travellers for contact tracing and necessary public heath measures |
| Identify an appropriate place for rapid health assessment and isolation in the event of detecting a potential ill passengers at PoEs |
| Ensure enough stocks of PPEs at PoEs and PoE staff trained on PPE use |
| Ensure ambulances services available or arranged as stand by status for 24 hours/7 days with health facilities designated for rapid referral of ill passengers and test the mechnims for safely trasnporting the ill passenger to health facilities designated |
| Identify ground services for environmental cleaning and disinfection at PoEs and ensure the cleaning and disinfection protocol for infectious disease has been put in place. |

| | |
|---|---|
| Joint contigency plan developed and tested at ground crossings between neighboring countries for public health emergencies based on joint risk assessment between the countries | |
| Identify health and non-health staff who could be mobilized to support response activities for any big outrbreak/pandemic such as border health, imigration and customs personnel, staff at migration reception, etcs and educate them for detection of any signs and symptoms and protocol for reporting them to pubic health authorities at PoEs and personal protection measures. | |
| **Country has updated inventory of the national strategic stockpile for public health emergency (medicines, vaccines, consumables, IPC and WASH and PPE supplies)** | |
| Country has developed Standard Operating Procedures for the management of logistics, including procurement, safe storage and delivery of supplies, both at the national and sub-national levels, prior to and during emergency | |
| The contingency plans for health sector contain a forcast of medical supplies and logistics to be required in line with scenarios of the contingency plan and specify roles and responsbility of transportation, storage and distribution in line with the national emergency preapredness and response plan/framework. | |
| Financial resources are allocated for identified essential supplies and equipment of health needs including for transportation, storage and distribution. | |
| Essential supplies and equipment for health sector response are pre-postioned at strategic locations considering transportation challenges as per country context during the emergency response | |
| **Country has contingency fund for emergency response for the health sector** | |
| Contingency funding channels for national, sub-national and local levels are clearly defined and understood by all relevant stakeholders | |
| Country has clear policy/protocol for cost of treatment/user fees including (lab tests, outpatient care, hospitalization, referral, medical exam and pharmaceuticals ) for emergency response, which is included in the contingency planning and disseminated amongst public and private facilities and the community | |
| **Health Facilities and their capacities and services (Private and Public) are mapped at all-levels (primary, secondary, tertiary, and outpatient).** | X |
| Country has reviewed and identified health facilities that are vulnerable to specific hazards (based on the country risk profile) | |
| Country has developed a risk mitigation strategy based on identified risks to hospitals and points of care | |
| Assessed risks at health facilities are prioritized and essential problems are mitigated and reduced | |

| | |
|---|---|
| Health facility maintainance staff is trained on mitigating the non - structural risks of the facility and regular resources are available for the risk mitigation. | |
| Health Facilities have risk communication protocols in place and key risk communication materials developed and updated regularly based on evolving situation and evidence based technical guidancce and available for use by all staff, patients, visitors and other stakeholders. | X |
| All health facilities including private sectors have a mechanism to ensure immediate reporting of probable and confirmed cases of all notifiable diseases to national surveillance system through their admistrative health authorities within 24 hours of case identification. | |
| All health facitities have a designated staff to collect, confirm and validate identified notifiable diseases with SoPs and forms for recording and reporting the cases identifed within 24 hours. | X |
| All legal procedures, administrative and financial mechanisms in place for emergency preparedness and response including procuring supplies and servcies. | |
| **Health Facilities have emergency contingency plans and Business Continuity Plans including maintaining continuity of services and operations during emergency response** | X |
| Health facilities have tested their contingency and business continuity plans (minimum once per year) | X |
| Health Facilities have identified the optimum number of staff (medical and non-medical) needed to ensure maintaining of essential services under Business continuity plan during the emergency response. | |
| Health workforce is well-trained on their roles and responsibilities during emergencies | |
| Systematic procedures in place to support the repurposing and reassignment of hospital staff (e.g. teleworking is available for staff with medical conditions who are at high risk for complications), with the hospital's corporate strategy defining roles and responsibilities for emergencies. | X |
| Health Facilities have an agreed, documented arrangement with the Ministry of Health or its equivalent to procure supplies, equipment and services necessary for the surge capacity. | |
| Surge roster of qualified human resources available and updated and includes the names and contact details of volunteers (e.g. retired staff, reserve military health personnel, senior medical and nursing students, community volunteers), with a back-up database of staff. | |
| Surveillance mechanism or procedures in place to moniotr health care associated infection and occupational hazards to esure safety of staff. | |
| HFs have all legal procedures for administration and financial mechanisms in place for procedures for procuring necessary supplies and services | |

| | |
|---|---|
| HFs have reserve stock for medical supplies and essential medicines required to activate BCP. | |
| HFs have an agreed, documented arrangement with the Ministry of Health or its equivalent to procure equipment necessary for the surge (e.g. mechanical ventilators, oxygen tanks) | |
| Procedures and plans in place to ensure management of the surge supply chain for essential medicines, diagnostics (including laboratory reagents, personal protective equipment and test kits ) and supplies for clinical care, therapeutic interventions and clinical management. | |
| **Infection Prevention and Control core capacities are in place within HFs** | X |
| A country strategy for an accelerated implementation of IPC core components is developed and put in place at the points of care | |
| Health Facilities have IPC committee and protocols with standard procedures for managing infectous cases in place and functional. | |
| IPC committee regularly conduct evaluation of transmission among health staff and moniotr compliance of IPC protocols among health care providers. | |
| All staff at health facilities are regularly trained on IPC protocols and to recognize and screen suspected infectious cases at their points of contact. | X |
| PPEs available and easily accessible to all hospital staff designated to interact with infectious cases | X |
| Designated isolation areas available for providing medical care to people with suspected, probable or confirmed infectious cases. | |
| Health Facilities have protocol in place for waste management including the management of biological and clinical waste. | |
| Mental health and psychosocial support services available for staff, their families and patients at health facities. | |
| SOP for mental health screening amongst infectious disease patients, their families and hospital staff ready and available in case there is a need to scale-up the emergency response. | |
| Have all hospital staff been trained in basic occupational safety and health measures and psychological first aid | |
| link to existing IBV assessments? | |
| | X |

| | |
|---|---|
| | |
| | |
| **The country has health workforce and surge capacity available and ready to respond to emergencies or outbreaks for protracted periods** | |
| Staff roster and surge capacity roster listing is available to mobilize workforce rapidly. Roster includes: contact information, skillset, availability | X |
| Emergency Medical Team(s) or equivalent are established, equipped and well-maintained to respond to an emergency in <24 hours | |
| Country has EMT cooridnation mechanisms in place to register and accredit EMTs and to coordinate EMTs deployment and performance moniotring through EMT CC (Coordination Cell) established at Ministry of Health or subnational health offices. | |
| SoPs and forms for registration, deployment, monitoring, reporting of EMT performances and activities through the EMT CC are develpoed and tested through simualtion exercises regularly. | |
| Country has necessary protocol or SoP developed and tested for deployment of international EMTs prequalified through WHO EMT project through EMT CC under MoH with WHO support. | |
| Rapid Response Team(s) RRTs or equivalent are established, equipped and well-maintained to support investigation and rapid assessment in <24 hours | |
| Surge teams have run a simulation exercise on 1 identified major risk to the country in <1 year | |
| **Country has existing mechanisms in place to signal and manage external emergency health workforce surge in case of emergency** | |
| SOPs or Policies/Procedures are in place to manage external workforce support for emergency response | |
| HFs have established mechanism to request additional health workforce resources to relevant health authorities in the event of an outbreak | |
| **Surveillance System is well-functioning with good surveillance coverage (completeness, promptness)** | |
| Case definitions, procedures for testing and reporting of reportable disease(s) are well-established with definitions available within health facilities (all-levels), inclusive of the private sector facilities | |
| Ensure EBS (Event Based Surveillance System) functional as part of EWARS in all hazard approach linking with community based surveilance and surveillance hub or PHEOC at national and subnational levels for triggering timely verification and investigation | |

For peer review only - http://bmjopen.bmj.com/site/about/guidelines.xhtml

| |
|---|
| Early Warning systems (all-hazards) are linked to the the country response mechanism (inclusive of health sector) such as Emergency Operation Center to promote quick decision-making and response measures through timely activation of the EoC. SoPs for EoC activation should be in place. |
| Weekly or month surveillance bulletins should be produced with regular surveillance data management and analysis at national and subnational levels. |
| Country adapt or operationalize a decsion instrument for assessment and notification of event which may constitute potential PHEIC to WHO into their routine surveillance data analysis and rapid risk assessment practice at national and subnational levels |
| National and local government ensures resources (human, financial and logistics) are planned to set up appropriate surveilance, reporting and early warning systems in essential sectors within 2-7 days after the emergency event (e.g. surveillances for water quality, food safety, hazard specific, IDP movements) |
| Country has mitigation strategy to ensure continuity of surveillance system during emergency response phase |
| **Country has capacity to investigate any signaled alert in <24 hours** |
| **Ensure trigers/criteria (alerts) for immediate investigation of any suspected outbreak are well established in the EWARS (Early Warning and Alert Surveillance) system** |
| Standard Operating Procedures (SOPs) are well-established to investigate alerts (inclusive of logistics, financing, mobilization of teams, etc.) including relevant authorizaton for investigations including food safety check and environmental sampling in restaurants and hotels, etc. |
| Country investigation teams (including rapid response teams) are well-trained and equipped to conduct field investigations |
| Ensure country has well trained multi-discplinary RRT teams which include relevant techncial expertise to address zoonosis, chemical, and radionulear hazards at appropriate levels in order to ensure their rapid depolyment when needed. |
| Ensure health facilities has trained staff and procedures for collection, packing and transportation of specimens to designated laboratories with preidentifed certified transportation agencies and budget planned in case of inability of RRT presence to the field. |
| Ensure dissemination of national guidelines for all health services personnel and medical practitiners on which specimen to be collected and where to send the specimens and how to protect them from getting infected. |

| **Mapping of Laboratories with diagnostic testing capacity available (tests performed, safety and security, material and equipment, and trained staff available)** |
|---|
| Reference laboratories have been identified for handling and testing of hazardous and highly infectious sample |
| Reference laboratories have sufficient staff trained, reagents and budget available to perform reference laboratoray roles for regional and local labs including training, supervision, QA/QC |
| Protocols available and posted in designated labs for specimen collection, packaging and shipment to the designated reference laboratory. |
| Coordination and collaboration mechanism established between labs at different health programs as well as other sectors such as university lab, private lab, labs at animal sectors |
| Laboratory personnel are well-trained on safety and security procedures for specimen collection, packaging, labelling, referral & shipment, including certification for the handling of infectious substances |
| Stand-by arrangements including MTA, Import permit and Expert permit in place with International Collaborating Centres/International Reference laboratories for confirmatory testing and with relevant air-lines to ship samples internationally. |
| Agreement or procedures established and practiced for epidemiological and virological information sharing (four way linking) between human and animal sector lab for detection of outbreak prone zoonotic diseases. |
| National reference laboratories maintain regular communications and collaborations with WHO collaborating centers and other internationally recognised public health laboratory networks for EQA and provision of lab reagents like primers, postive control, etc. for detecting new infectious diseases. |
| Conduct Simulation exercises including drills frequently to test mecahnism or interoperablility for alert detection, test and response between relevant sectors such as surveillance, RRT and lab sectors. |
| Ensure laboratory data sharing mechanism or database is well established with refering health personnel or faciliites, surveillance, and public health authorities for uniform data flowing and reporting. |
| All laboratories in the country which deal with infectious hazards have their relevant staff trained for bio safety and security, enough stock of PPEs. |
| All laboratories in the country which deal with infectious hazards have enough budget to perform their normal duties as well as contingency fund planned or identified for surge need and reflected in the contigency plans |

| |
|---|
| Integrated health information system is in place and functional to ensure data on case and death are recorded and reported through one data application between health facilities (hospitals, clinic both of public and private), surveilance system, lobatories and health authorities from community to central levels. |
| Rapid Health Assesement tool for health sector is in place for use at the intial stage of the emergencies and technical team identfied and trained for the assessment. |
| Link with country health information system to obtain secondary health information at the areas affected by emergencies, including immuninzation coverage rate, disease profiles and endemicity (vector borne, water borne and food borne diseases, etc.) |
| Relevant staff trained on production and distribution of various information products such as situation report, health cluster/sector bulletin, outbreak newsletters, and templates or formats for those infromation productions are standardized and ready for use. |
| Simplified operational tool for reporting of 4 Ws (Who is doing what, where and when) is standardized and used among all stakeholders and partners to support coordination and decision making |
| Post diaster need assessment tool for health sector is in place for early recovery planing and actions in coordination with other sectors. |
| **Country has mapped key community stakeholders and leaders within communities (administrative, traditional, religious leaders; professional associations, youth groups, school leaders, etc.)** which will govern community emergency preparednessa and response in coordination with local government health authority. |
| Ensure local government authorities undertake risk analysis of communities incluidng vulnerablility and capacities to develop community preapredness and response plans with hazard prone areas and high risk populations identifed. |
| Ensure the community plans are developed with participatory and inclusive approach of all stakeholers and partners and also link with nearest health facility or post. |
| Ensure that community based activities are incoporated into national and local, subnational response plans and engage networks of community service providers (including NGOs, private health care providers and volunteers to support response efforts in a coordinated manner. |

For peer review only - http://bmjopen.bmj.com/site/about/guidelines.xhtml

| |
|---|
| Communities have developed preparedness and response plans based on community needs and norms (including family and community preparedness) and tested the plans at least once a year by ensuring the interoperability with health sector preapredness plans of the local health government. |
| **An appropriate community surveillance and monitoring strategy has been developed and is in place to detect rumors and relevant health information of the population not accessing facility-based health services** |
| Establish or Strengthen community based surveillance system with engagement of community health workforce to support health sector EWARS ( Event Based surveillance system) in all hazard approach |
| Social media communication strategy is developed to address the rumors and myths on social media (e.g. Facebook, Weibo, Twitter, etc.) with evidence-based engagement with users |
| Sufficient communication materials have been tested and are available to deliver key health messages |
| Health sector has designated and trained key spokesperson(s) for media communication and health information dissemination |
| Health sector is linked to overall emergency response communications mechanism to harmonize messaging during disaster |
| **Community volunteer mechanisms are well-established, mapped, and functional to identify, train and maintain volunteers for initial life saving and emergency response activities.** |
| **Community-based health system is well-mapped and described** |
| Create or leverage existing database of community health workforce with different skills and qualified workers including unempolyed and retired workers and these are updated regularly and kept with local health authorities or primary health care centers. |
| Leadership and lines of communication within the community-based health system and health surveillance are well-established and functional at the national, subnational and local levels |
| Ensure local government or primary health care centers have mapping of networks of community service providers and coordinate with and provide resources for community goverance committees or community leaders in organizing and providing community health services in line with the commumty service protocol established in a context relevant ways. |
| Country has emergency hotline available for persons to call during an emergency with key information |

| |
|---|
| Community feedback mechanism is well-established and results are well-linked to emergency response leadership and coordination mechanisms and infodemic management |
| Trainings regularly conducted for community health worforces with development of standard training package on life saving interventions including first aid and mass casulty care, public health interventions by trainers from local and national health authorities in close coordination with commuity governing committees and leaders |
| Ensure basic supplies and equipment such as first aid kit and health emergency kits are stocked at communities or local health authorities to provide them immediately after the emergency event |
| Consider to implment  a mechanism to recognize and remunerate the communty workforce supporting with non-payment incentives or payment in coordination with stakeholders and partners as per local context and culture |
| Ensure safety and health of community health workforce by providing PPEs approprite to the tasks performed, protecting against violence and harassment and offering psychosocial support |
| Develop and disseminate SoPs for Infection Prevention and Control that include community health workforce and ensure through training for all users of standard and additional (transmission based) IPC precautions including PPE use. |
| Develop a medical supply provision and distribution mechanism appropriate to each communty context in coordination with local health authorities to ensure uninterrupted medical supplies during the emergency situations |
| Communities have developed response plans based on community needs and norms (including family and community preparedness) |
| **The country's supply chain and movement of supplies and personnel is well-mapped and functional** |
| There is demonstrated capacity within the country to support movement of staff and medical supplies |
| There is demonstrated country capacity to produce a regular gap analysis of the required stock at the sub-national level and local district level |
| There is adequate storage and warehousing for PPE and other medical supplies in support of a scaled-up emergency response |

| |
|---|
| |
| **WHO Country Office has developed and implemented Business Continuity Plan and accompanying Action Plan to maintain critical operations during emergency response** |
| WCO has pre-identified the key 6 IMST staff that are well-trained and understand roles and responsibilities |
| WHO has a roster of technical and non-technical expertise to draw from in case of need during emergency response |
| WHO has in-country capacity to conduct a rapid risk assessment <48 hours |
| **WHO is well-integrated in the Country emergency preparedness and response coordination mechanisms (as appropriate) for the health sector** |
| WHO is integrated in the UN coordination mechanism in-country |
| WHO has established links with the Country for information sharing including but not limited to IHR-relevant information |
| Country and WHO have pre-identified technical areas where WHO can support the Country in emergency response |
| **Current partner capacity/activities and potential expansion of activities in the event of an emergency are well-mapped through the 4W Matrix** |
| Partners are included and regularly participate in health sector and cluster meetings |
| Partners have been included in planning processes (response plan, contingency planning)and simulation exercises |
| Country has established and disseminated regulations and procedures for partners (i.e. certification, validation of health staff licensing, visas for external staff, etc) |
| Risk assessments and repository of information (survey, assessment, etc) exists and is well-functioning |
| |

Few thoughts:
Potentially look at PIP Framework/Lab for potential opportunities for cross-walking of actions
Not much in this draft for novel disease actions and readiness (but potentially you wouldn’t nee

*Example: During a risk ranking exercise, Country X identified seasonal flooding as a major risk in*
- *Applied the flood checklist at the national and within the localized area(s) mo*
- *Reviewed the most recent simulation exercise results for flooding response*
- *Compiled results of the most recent After-Action Review (and assessed the st*

- *Reviewed existing flood contingency plans*
- *Reviewed health facility capacities and resilience to flooding*
- *Assessed flood mapping with Ministry of Environment to identify vulnerable f*
- *Verified stockpiles and positioning of medical supplies*
- *Incorporated feedback from national experts, partners and other relevant pa*

For peer review only

| Additional items/ actions to the framework | Burkle et al 2011 | Additional items/ actions to the framework |
| --- | --- | --- |
| Staff wellfare | | N/A |
| Availability of critical items | | |

X

For peer review only

For peer review only

For peer review only

For peer review only

For peer review only

For peer review only

X

X

X

X

For peer review only

For peer review only

For peer review only

For peer review only

X

For peer review only

X

X

X

For peer review only

d so much details as we are in a special situation)

*the next year. Because flooding was identified as major risk that is likely to occur in the next year, t*
*ost likely to be affected by flooding to identify strengths and potential gaps*

*atus of key actions recommended)*

For peer review only

*flood areas/communities and any potential disruption to health services*

*rties on current capacities and gaps to respond*

For peer review only

For peer review only

For peer review only

For peer review only

For peer review only

For peer review only

For peer review only

For peer review only

For peer review only

For peer review only

For peer review only

For peer review only

For peer review only

*:he country assessed its baseline readiness for the health sector to respond to flooding by:*

For peer review only

For peer review only

For peer review only

For peer review only

For peer review only

For peer review only

For peer review only

For peer review only

For peer review only

For peer review only

For peer review only

For peer review only

For peer review only

For peer review only

For peer review only

| Author | Year | Name of model |
|---|---|---|
| Burkle et al | 2011 | Health and Human Services Medical Surge Capacity and Capability (MSCC) framework |
| Burkle et al | 2011 | Multiagency coordination center (*MAC*) model |

For peer review only

| Purpose/ use |
|---|
| Illustrates the role and placement of healthcare coalitions (Tier 2) in the overall federal, regional, state, and local response structure |
| Representation of a multiagency coordination approach used for regional planning |

For peer review only

| URL link |
|---|
| https://journals.lww.com/pccmjournal/_layouts/15/oaks.journals/ImageView.aspx?k=pccmjournal:2011:11001:00006&i=F2-6&year=2011&issue=11001&article=00006&type=Fulltext |
| https://journals.lww.com/pccmjournal/_layouts/15/oaks.journals/ImageView.aspx?k=pccmjournal:2011:11001:00006&i=F3-6&year=2011&issue=11001&article=00006&type=Fulltext |

For peer review only

BMJ Open

# Defining and identifying critical elements of, and lessons learned from addressing, ‘operational readiness’ for public health emergency events, including COVID-19: a rapid scoping review protocol

| | |
|---|---|
| Journal: | *BMJ Open* |
| Manuscript ID | bmjopen-2021-060526.R1 |
| Article Type: | Protocol |
| Date Submitted by the Author: | 06-Aug-2022 |
| Complete List of Authors: | English, Rene; Stellenbosch University Faculty of Medicine and Health Sciences, Global Health<br>Nyasulu, Juliet; Stellenbosch University, Faculty of Medicine and Health Sciences, Global Health Faculty of Medicine and Health Sciences Division of Health Systems and Public Health; AFRIQUIP, Health Systems Strengthening<br>Berner, Karina; Stellenbosch University Faculty of Medicine and Health Sciences, Rehabilitation Sciences<br>Geduld, Heike; Stellenbosch University, . Division of Emergency Medicine, Department of Family and Emergency Medicine, Faculty of Medicine and Health Sciences<br>McCaul, Michael; Stellenbosch University Faculty of Medicine and Health Sciences, Department of Interdisciplinary Sciences; Stellenbosch<br>Joseph, Conran; Stellenbosch University, Division of Physiotherapy, Department of Health and Rehabilitation Sciences,<br>Pappin, Michele; Stellenbosch University, Faculty of Medicine and Health Sciences, Global Health Faculty of Medicine and Health Sciences Division of Health Systems and Public Health<br>Gobat, Nina; World Health Organization<br>Boulanger, Linda; WHO<br>Louw, QA; Stellenbosch University, Physiotherapy; Stellenbosch University, |
| <b>Primary Subject Heading</b>: | Infectious diseases |
| Secondary Subject Heading: | Emergency medicine, Infectious diseases, Public health |
| Keywords: | INFECTIOUS DISEASES, PUBLIC HEALTH, ACCIDENT & EMERGENCY MEDICINE |
| | |

SCHOLARONE™
Manuscripts

# Defining and identifying critical elements of, and lessons learned from addressing, 'operational readiness' for public health emergency events, including COVID-19: a rapid scoping review protocol

**Word Count: Main document:** 3505 **Abstract :** 298

For peer review only



For peer review only - http://bmjopen.bmj.com/site/about/guidelines.xhtml

# Abstract

**Introduction:** Much is known around public health preparedness and response phases. However, between the two is phases is operational readiness which comprises the immediate actions needed to respond to a developing risk or hazard. Currently emergency readiness is embedded in multiple frameworks and policy documents related to the health emergency cycle. However, knowledge about operational readiness' critical readiness components and actions required by countries to respond to public health eminent threat is not well known. Therefore, we aim to define and identify the critical elements of 'operational readiness' for public health emergencies, including COVID-19, and identify lessons learnt from addressing it, to inform the World Health Organisation (WHO) Operational Readiness Framework.

**Methods and analysis:** This is a scoping review following the Joanna Briggs Institute guidance. Reporting will be according to the PRISMA Extension for Scoping Reviews (PRISMA-ScR) checklist. MEDLINE, Embase and Web of Science databases and grey literature will be searched and exported into an online systematic review software (e.g. Rayyan in this case) for review. The review team, which apart from scoping review methodological experts include content experts in health systems and public health and emergency medicine, prepared an a priori study protocol in consultation with WHO representatives. ATLAS.ti V9 will be used to conduct thematic data analysis as well as store, organise, and retrieve data. Data analysis and presentation will be carried out by five reviewers.

**Ethics and Dissemination:** This review will reveal new insights, knowledge and lessons learnt that will translate into an operational framework for readiness actions. In consultation with WHO, findings will be disseminated as appropriate (e.g., through professional bodies, conferences, and research papers). No ethics approvals are required as no humans will be involved in data collection.

**Protocol registration:** This rapid scoping review has been registered on Open Science Framework (doi:10.17605/OSF.IO/6SYAH).


For peer review only

## Study strengths and limitations

- The Covid-19 pandemic has shown that globally, countries even with well-resourced health systems and structured emergency preparedness plans in place were not able to sufficiently respond to the threat. Meaning that gaps existed between transition from preparedness to responding which is readiness. Therefore, defining and identifying critical elements of operational readiness for public health emergency events, including COVID-19 is critical
- Currently emergency operational readiness is embedded between preparedness and response and in most cases poorly defined. Therefore, we believe that an understanding of health systems readiness in responding to emergency is key.
- The review team included members that provided a mix of methodological and content expertise that will aid decisions regarding a speed-rigour trade-off.
- Currently there is no clear definition of activities that constitute health systems emergency readiness and people use different names whilst others name it either preparedness or response. In case these operational readiness definition words are not captured in the scoping review search strategy, this will be a limitation.
- Limiting the search to English full texts and last 11 years may lead to and publication timeline biases

For peer review only

## Introduction

Much has been documented about how countries should best prepare to respond to health emergencies (1–3). The effectiveness of ‘readiness’ – a concept referring to actions needed to rapidly respond to an imminently anticipated risk or hazard – largely depends on the sufficiency and comprehensiveness of prior longer-term ‘preparedness’ policies (4). However, little is known about the critical components of readiness and the kinds of readiness actions that should be taken by countries at all levels in response to health emergencies. Such knowledge is critical to inform operational readiness actions for future events.

Health Emergencies and Disaster Risk Management (Health-EDRM) encompasses the intersecting fields of emergency and disaster medicine, health systems strengthening and resilience, disaster risk reduction, humanitarian response and community health resilience. Within this framework, it is accepted that the management of emergencies is a whole-of-society approach, focusing on all hazards and involving multiple sectors and multiple disciplines (5). Health-EDRM involves four broad components, namely (i) hazard vulnerability assessment (HVA) and mitigation; (ii) preparedness; (iii) response and (iv) recovery. Within these, the activities of ‘readiness’ will occur within both HVA and mitigation and preparedness components. These readiness activities are linked both temporally and structurally to a specifically identified hazard, whether that is an infectious disease, or climate change event. Thus, what constitutes ‘readiness’ is determined by the nature of the hazard.

The World Health Organisation (WHO) Strategic Framework for Emergency Preparedness (6) is a unifying framework for country-level public health emergency preparedness. This framework describes operational readiness to respond to emergencies as a continuous, co-ordinated process, involving a multisectoral response, incorporating multiple level infrastructure, and following an all-hazard approach with a focus on high priority risks (6).

The current COVID-19 global pandemic has exposed the fragility of health systems to respond to shocks in the form of disease outbreaks or health emergencies (7). According to the WHO, the response of a public health system to an outbreak or health emergency such as the COVID-19 pandemic can be defined as a cycle that sways between preparedness and the actual response. Through applying a governance lens, the WHO has developed an Emergency Response Framework (4), which describes the stages of an outbreak or health emergency. As alluded to above, readiness to respond lies somewhere between preparedness and response; it is the instant action to an emergent or prominent risk and is hugely reliant on adequate preparedness (4). In many instances, implementation of these well-designed disaster preparedness policies is met with significant challenges due to flaws in the ‘readiness’ of systems to do so. ‘Readiness’ as a concept has not been fully designed, and therefore it is critical to define the critical components of readiness and the types of readiness actions to be taken in response to outbreaks and health emergencies to inform operational readiness actions for future events (8). A preliminary search of MEDLINE, the Cochrane Database of



For peer review only - http://bmjopen.bmj.com/site/about/guidelines.xhtml

Systematic Reviews, Prospero and JBI Evidence Synthesis revealed no current or underway systematic reviews or scoping reviews on the topic. The WHO is currently developing an Operational Readiness Framework intended to guide effective action. Specifically, the purpose of the framework is to scale-up preparedness for a specific risk at the local and national levels by considering how ready a country is to respond to the imminent threat, and to identify key actions needed to be ready to respond effectively to that threat. To this end, WHO has called for a rapid scoping review to be conducted that will assist with defining available evidence related to readiness and readiness actions.

## Aim and objectives

The overarching aim of this rapid scoping review is to define and identify the critical elements of 'operational readiness' for public health emergencies, including COVID-19, and identify lessons learnt from addressing it, to inform the WHO Operational Readiness Framework.

To this end, the following objectives will be addressed:

1. To conceptualise and define 'operational readiness'.
2. To map and describe frameworks, policies and evidence/information related to 'operational readiness' for all hazards, with a strong focus on infectious diseases.
3. To define critical elements of 'operational readiness' at multiple levels of the health system (community, local, sub-national, national, regional, global).
4. To identify lessons learned from enhancing or influencing 'operational readiness' (at multiple levels).

## Review question

### Primary scoping review method question

The primary review question was formulated using the PCC (Population, Concept and Context) method (9): *How can/do communities/countries/regions/global institutions operationalise readiness for imminent public emergencies?*

### Sub-questions

The review will seek to answer the following additional or sub-questions:

1. How is 'operational readiness' for public health emergencies conceptualised and defined?
2. What are the critical elements (dimensions, operational actions, coordination) of 'operational readiness' for public health emergencies at multiple levels (community, local, sub-national, national, regional, global)?
3. How did countries ready/ prepare for COVID-19?



For peer review only - http://bmjopen.bmj.com/site/about/guidelines.xhtml

4. What lessons have been learned about 'operational readiness' during for example, COVID-19/ Ebola, with a strong focus on infectious disease emergencies?

# Keywords

All hazards; Disaster planning; Epidemic; Imminent threat; Infectious diseases; Outbreak; Pandemic; Public health emergency

# Eligibility criteria

## Inclusion criteria

### *Participants/ population*

These are the groups or organisations who would respond and/or lead the response, and include the following:

- Communities (local, subregional, or national level)
- National, country, regional and global governments
- Global health organisations, such as the WHO

### *Concept*

The purpose of the scoping review is to define 'operational readiness. This concept refers to the immediate action(s) that are taken to pre-position response actions needed to address a proximal, imminent hazard/ threat – such as an 'acute' infectious disease outbreak or natural disaster threat (an all-hazards approach). These include but not limited to Disease Outbreaks, epidemics/ or pandemics, public health emergency, communicable diseases, Incident Management System, country risk profile and many other details. The concept lies between 'preparedness' and 'response'. To find evidence of readiness interventions, we will look at sources referencing preparedness, planning and disaster management as the term 'readiness' may be embedded in 'preparedness' – or the term 'preparedness' may be used to describe actions that (based on our definition) we would describe as readiness.

We will consider sources that:

- Conceptualise, theorise, define, or describe or interpret 'operational readiness' and/or preparedness for public health emergencies (at community, country, regional or global levels) at the time when the threat of an infectious disease outbreaks or natural disaster becomes known, within a specific timeframe (viz., defining 'imminence').



For peer review only - http://bmjopen.bmj.com/site/about/guidelines.xhtml

- Contain explanations, descriptions, intervention approaches, analysis or frameworks or anticipatory actions for 'operational readiness' or preparedness for public health emergencies (at community, country, regional or global levels) when the threat of an infectious disease outbreaks or natural disaster becomes known.
- Provide the nature and description of critical elements (dimensions, coordination, roles of key stakeholders such as the community, health actors, policy makers etc.) of 'operational readiness' for public health emergencies at community, national, regional, and global levels.

### *Context*

The context of health emergencies refers to natural disasters and infectious disease threats (new and re-emerging) – i.e., all hazards. Important to note is that these threats are acute (imminent) and impact the health of populations. These health emergencies occur within the community as well as health system and health service contexts.

The proposed definition of a 'health emergency' is an extraordinary event that is determined to 'constitute a public health risk whose scale, timing, or unpredictability threatens to overwhelm routine capabilities of the health system' (10 pS9) and potentially require a coordinated response at multiple levels (10,11).

### *Types of sources*

- Peer-reviewed review or empirical research (any study design) that is available in full-text and published in scientific journals between 2010 and 2021.
- Publicly available policy frameworks and programme reports.
  Published conference reports or electronic theses.
- Documents of which the full text or abstract is available in the English language. If the English version of the abstract is potentially eligible for inclusion, the full text (if German/ French/ Afrikaans) will be translated to make a final decision on eligibility.

### Exclusion criteria

- Papers focusing exclusively on longer term preparedness actions or exclusively on response actions will be excluded.
- Papers reporting on contexts beyond health emergencies or not focused on disease prevention and control will be excluded.

## Methods and data analysis

This rapid scoping review will be conducted in accordance with the Joanna Briggs Institute (JBI) methodology for scoping reviews (9). The review will be reported using the PRISMA Extension for Scoping Reviews (PRISMA-ScR) (12) and PRISMA-S Extension for Searches in Systematic Reviews (13).



For peer review only - http://bmjopen.bmj.com/site/about/guidelines.xhtml

## Search strategy

The search strategy will aim to locate peer-reviewed review or empirical research (any study design) that is available in full-text and published in scientific journals, publicly available policy frameworks, programme reports, and published conference reports or electronic theses. This will include humanitarian literature where health impacts or effects are the focus. Due to the rapid nature of the scoping review, we will restrict the search to studies published between 2010 – 2021 and those available in English (potentially eligible Afrikaans, German or French full texts, according to the English abstract, will be translated into English).

The electronic databases to be searched include MEDLINE, Embase and Web of Science. An initial limited search of MEDLINE was undertaken to identify articles on the review topic. The text words contained in the titles and abstracts of relevant articles, and the index terms used to describe the articles were used to draft a full search strategy for MEDLINE. The search strings and terms were developed iteratively and in consultation with the WHO and are centred around three key concepts: (i) emergencies/ diseases/ natural disasters; (ii) readiness/ preparedness/ risk/ planning; and (iii) health systems/ community. The search strategy, including all identified keywords and index terms, was subsequently adapted for Embase and Web of Science. Searches will be conducted by an expert information specialist in consultation with the review team. The reference list of all included sources of evidence will also be screened for additional studies. Reporting of the searching will be guided by the PRISMA-S Extension for Searches in Systematic Reviews (13).

### *Searching other resources*

Sources of unpublished studies/ grey literature to be searched include various targeted repositories, websites, and databases. These include global organisations (e.g., the WHO, United Nation Children's Fund [UNICEF], United Nations Office for Disaster Risk Reduction [UNDRR], United Nations International Strategy for Disaster Reduction [UNISDR], International Federation of Red Cross [IFRC], International Committee of the Red Cross [ICRC]), regional WHO offices (i.e., Southeast Asian, African, Western Pacific, Pan American, European and Eastern Mediterranean) and the European Centre for Disaster Medicine (CEMEC). Societies and organisations include the World Association for Disaster and Emergency Medicine (WADEM), Médecins Sans Frontières (MSF) and ReliefWeb. National websites include the United States Centres for Disease Control and Prevention (CDC) and Federal Emergency Management Agency (FEMA), the Robert Koch Institute (RKI), Public Health England. Lastly, Evidence Aid will be included as an evidence repository.

## Selection of studies

All search hits will be imported into Rayyan V0.1.0 software (Rayyan Systems Inc., MA, USA) (14) for screening, checking of duplicates and selection of final documents to be included. To support consistent abstract and title screening and refine eligibility, senior authors (RE, HG and



For peer review only - http://bmjopen.bmj.com/site/about/guidelines.xhtml

MM) together with the title and abstract screeners (MP and MYC), will (as an initial step) independently and in duplicate screen 100 articles, followed by discussion. The following proposed screening approach is adapted from the Cochrane Rapid Reviews Methods Group guidance for systematic reviews to balance rigour and speed consistent with rapid reviews (15,16). Twenty percent of titles and abstracts will be screened by two reviewers (MP and MYC), independently, in duplicate and with conflict resolution, to remove obviously irrelevant reports. After this, one reviewer (MP) will screen the remaining titles and abstracts while the second reviewer (MYC) will verify excluded titles and abstracts and resolve conflicts (15). If required, a third senior reviewer (HG or RE) will resolve any disagreements The full texts of selected citations will subsequently be assessed in detail against the eligibility criteria by the first reviewer, while the second reviewer will verify all excluded full texts (15). Reasons for exclusion of sources of evidence at full text that do not meet the inclusion criteria will be recorded. This information will be reported and added to a table of excluded studies in the scoping review. Any disagreements that arise between the reviewers at each stage of the selection process will be resolved through discussion, including with an additional senior reviewer (HG or RE) if needed. If study eligibility is unclear owing to missing data, further information will be requested from study authors. The results of the search and the source inclusion process will be reported in full in the final scoping review and presented in a PRISMA-ScR flow diagram (12).

## Data extraction and management

Due to the rapid design and potential large pool of included studies we will use a dynamic approach to data extraction and management. For an included study yield of ≤25, data extraction will be done by one reviewer (MP), while a second reviewer (KB or MYC) will check for completeness and accuracy (15). For yields between >25 but ≤75, two or more extractors will be used (e.g., MP/KB/MYC/CJ/QL/RE), while an additional reviewer will check for correctness and accuracy (17). In the case of more than 75 included sources, we will consider a prioritisation process whereby we rank or stratify studies based on design and relevancy to the scoping review. Prioritised studies will then be included for data extraction until the review team, together with WHO, agrees that data saturation has been achieved. The reviewers will discuss the nature of the information that will be extracted before commencing the process to facilitate coherence. Any uncertainties before and during the extraction process will be discussed with team members to make a final decision.

The data extracted will include author name(s), publication year, publication country and World Bank classification, source classification as primary/ secondary/ multi-method, publication type, study design, aim/ purpose, sample/ facility description, method/ tool for data collection, modifications to the data tool (if any), level (community, national, etc.), type of emergency, operational readiness definition, preparedness definition, key actors, challenges/ recommendations, lessons learnt, and other relevant information/ conclusions. In addition,



For peer review only - http://bmjopen.bmj.com/site/about/guidelines.xhtml

data regarding readiness will be extracted according to the WHO's operational readiness components – these include:

- Leadership, governance, and coordination,
- Country risk profile,
- Operational planning and coordination,
- Contingency finance,
- Health facility capacity and service delivery,
- Health workforce/ human resources,
- Early warning or surveillance and health information systems,
- Community resilience and risk communications,
- Logistics or supply chain for access to essential medicines,
- WHO readiness,
- Partner readiness.

Framework details and any associated actions will be recorded. Finally, information regarding relevant models will be extracted, including URL links to figures/ diagrams.

A draft extraction form will be pilot-tested independently by two reviewers using a sample of two to three potential included full-text articles/ evidence sources (17). Based on feedback from the two reviewers, the form may be modified and revised as necessary during the process of extracting data from each included evidence source (17). Necessary modifications will first be discussed within the review team for consensus, and any changes implemented will be reported in the final scoping review. Authors will be contacted where possible to clarify or obtain additional information.

## Methodological appraisal

Included peer-reviewed literature will be evaluated for quality based on appropriate pre-existing methodological quality checklists.

## Data analysis and presentation

Data will be synthesised in line with the core objectives of the rapid scoping review.

The included documents will be analysed using qualitative thematic analysis through an deductive synthesis approach (18–20). We are proposing to use ATLAS.ti V8 (Scientific Software Development GmbH) (https://atlasti.com/) to conduct thematic data analysis as well as store, organise, and retrieve data. Data analysis will be carried out by the project group researchers, who have vast knowledge and experience in undertaking reviews, including scoping reviews, that have used qualitative thematic analysis.

Findings will be deductively coded into a conceptual model that is taken from the WHO Country Readiness for Health Checklist to define and identify the critical elements of



For peer review only - http://bmjopen.bmj.com/site/about/guidelines.xhtml

'operational readiness' for public health emergencies, including COVID-19, and identify lessons learnt from addressing it. We will also identify if there are additional consistent themes emerging from the analysis that are not currently included in the WHO Checklist, as potential additional items.

The analysis will start by evaluating documented text line-by-line, allocating text a descriptive label and code. The same will be done for the other focused questions on understanding the similarities and differences between operational readiness and preparedness and identifying critical elements. The researchers will remain close to the data from the primary sources when defining and understanding the meaning structure of these concepts and phenomena. Since the conceptual understanding of 'operational readiness' and 'preparedness' will be initially explored, described, and theorised and may vary across sources, we will initially use broad, higher order codes (which may form main themes) developed deductively from the framework to organise the data. Once all data have been initially coded and collated, all the potentially relevant coded data extracts will be sorted and collated into themes and sub-themes (including a 'miscellaneous' theme for codes that do not clearly fit into existing themes (20). Senior reviewers (RE, HG and QL) will debrief the researchers primarily responsible for the thematic analysis, and the review team will meet regularly to discuss codes and themes, including potential merging or further break-down of themes (depending on whether there are enough data to support a theme, or the data are deemed too diverse). The themes will represent the synthesis and interpretation that go beyond the primary sources as well as deliver new insights and knowledge, which will translate into an operational framework for readiness and important lessons learnt.

A numerical description of the extent and nature of included evidence sources will be presented using tables and charts, accompanied by narrative summaries to describe how the results relate to the review's objectives.

## Patient Public involvement

Patients or the public were not involved in the design, or conduct, or reporting, or dissemination plans of our research

## Ethics, reporting and dissemination

No ethical approval is needed for this rapid scoping review, given that included sources will comprise of published and publicly available information.

The study was expected to commerce in December 2021 to July 2022 with first scientific publication output expected in August 2022. The Stellenbosch University (SU) review team will work with the WHO commissioning group and draw on the expertise of expert advisors to the review team to produce the following outputs. Weekly internal and SU-WHO meetings have been conducted to provide input into the development of this research protocol and will



For peer review only - http://bmjopen.bmj.com/site/about/guidelines.xhtml

continue to aid understanding of emerging insights and findings that can inform work tasks relevant to the technical product development. Interim findings from the rapid scoping review will be presented to the WHO. Following feedback, an updated interim report incorporating feedback from the WHO and expert advisory team will be presented. The final report of the full rapid scoping review will be delivered, along with a PowerPoint presentation to the WHO commissioning group of findings with talking points. In consultation with the WHO, findings will be disseminated further as appropriate (e.g., through professional bodies, conferences, and research papers). By defining evidence related to critical readiness components and actions, this review will reveal new insights, knowledge and lessons learnt that will translate into an operational framework for readiness actions.

## Acknowledgements

The rapid scoping review was commissioned by the WHO to inform an Operational Readiness Framework for the Country Readiness Strengthening Department in the World Health Emergencies Program in WHO (Reference #: 2021/1145765; Unit: MST; Cluster: QNF/SCI).

## Authors' contribution

RE is the Principal investigator and JCYN coordinates the team, the research process and is a corresponding author. NG and LB contributed to content through the process, provided guidance on the expected outcomes and WHO guidelines to be followed. KB and MP drafted the original protocol, MM, QL, CJ and HG provided technical guidance on the methodology. All authors were involved in the conceptualization of the concept paper and proposal writing and read the final version of the submitted protocol.

## Funding

This work is supported by the World Health Organization through a consultancy fee and not research grant [Reference: APW/RR/Readiness/2021/1145765], as a result, there is no grant number

## Conflicts of interest

There is no conflict of interest in this project.

For peer review only

## References


1. Savoia E, Lin L, Viswanath K. Communications in public health emergency preparedness: A systematic review of the literature [Internet]. Vol. 11, Biosecurity and Bioterrorism. Mary Ann Liebert, Inc.; 2013 [cited 2021 Oct 4]. p. 170–84. Available from: /pmc/articles/PMC3778998/

2. Tang R, Fitzgerald G, Hou XY, Wu YP. Building an evaluation instrument for china's hospital emergency preparedness: A systematic review of preparedness instruments [Internet]. Vol. 8, Disaster Medicine and Public Health Preparedness. Lippincott Williams and Wilkins; 2014 [cited 2021 Oct 4]. p. 101–9. Available from: https://pubmed.ncbi.nlm.nih.gov/24622400/

3. Ejeta LT, Ardalan A, Paton D. Application of behavioral theories to disaster and emergency health preparedness: A systematic review. PLoS Curr. 2015 Jul;7(DISASTERS).

4. WHO Emergencies Preparedness Team. Emergency response framework [Internet]. 2nd ed. World Health Organization, editor. Geneva; 2017. Available from: https://www.who.int/publications/i/item/9789241512299

5. Lo STT, Chan EYY, Chan GKW, Murray V, Abrahams J, Ardalan A, et al. Health Emergency and Disaster Risk Management (Health-EDRM): Developing the Research Field within the Sendai Framework Paradigm. Int J Disaster Risk Sci [Internet]. 2017 Jun 1 [cited 2021 Oct 4];8(2):145–9. Available from: www.ijdrs.comwww.springer.com/13753

6. WHO Emergencies Preparedness Team. A strategic framework for emergency preparedness [Internet]. World Health Organization, editor. Geneva; 2016. Available from: https://www.who.int/publications/i/item/a-strategic-framework-for-emergency-preparedness

7. Haldane V, De Foo C, Abdalla SM, Jung AS, Tan M, Wu S, et al. Health systems resilience in managing the COVID-19 pandemic: lessons from 28 countries [Internet]. Vol. 27, Nature Medicine. Nature Research; 2021 [cited 2021 Oct 4]. p. 964–80. Available from: https://pubmed.ncbi.nlm.nih.gov/34002090/

8. Khan Y, O'Sullivan T, Brown A, Tracey S, Gibson J, Généreux M, et al. Public health emergency preparedness: A framework to promote resilience. BMC Public Health [Internet]. 2018 Dec 5 [cited 2021 Oct 4];18:1344. Available from: https://bmcpublichealth.biomedcentral.com/articles/10.1186/s12889-018-6250-7

9. Peters M, Godfrey C, McInerey P, Munn Z, Tricco A, Khalil H. Chapter 11: Scoping Reviews (2020 version). In: Aromataris E, Munn Z, editors. JBI Manual for Evidence Synthesis [Internet]. JBI; 2020. Available from: https://synthesismanual.jbi.global

10. Nelson C, Lurie N, Wasserman J, Zakowski S. Conceptualizing and defining public health emergency preparedness. Vol. 97 Suppl 1, American journal of public health. American Public Health Association; 2007. p. S9–11.

11. World Health Organization. Chapter 11: Public health emergencies. In: Advancing the




For peer review only - http://bmjopen.bmj.com/site/about/guidelines.xhtml

right to health: the vital role of law [Internet]. Geneva: World Health Organization; 2017. Available from: https://apps.who.int/iris/handle/10665/252815

12. Tricco AC, Lillie E, Zarin W, O'Brien KK, Colquhoun H, Levac D, et al. PRISMA Extension for Scoping Reviews (PRISMA-ScR): Checklist and Explanation. Ann Intern Med [Internet]. 2018 Oct 2 [cited 2019 Apr 8];169(7):467–74. Available from: http://annals.org/article.aspx?doi=10.7326/M18-0850

13. Rethlefsen ML, Kirtley S, Waffenschmidt S, Ayala AP, Moher D, Page MJ, et al. PRISMA-S: an extension to the PRISMA Statement for Reporting Literature Searches in Systematic Reviews. Syst Rev [Internet]. 2021 Jan 26 [cited 2021 Oct 4];10(1):39. Available from: https://doi.org/10.1186/s13643-020-01542-z

14. Ouzzani M, Hammady H, Fedorowicz Z, Elmagarmid A. Rayyan -a web and mobile app for systematic reviews. Syst Rev [Internet]. 2016 [cited 2021 Oct 4];5(1):210. Available from: http://systematicreviewsjournal.biomedcentral.com/articles/10.1186/s13643-016-0384-4

15. Garritty C, Gartlehner G, Nussbaumer-Streit B, King VJ, Hamel C, Kamel C, et al. Cochrane Rapid Reviews Methods Group offers evidence-informed guidance to conduct rapid reviews. J Clin Epidemiol [Internet]. 2021 Feb 1 [cited 2021 Oct 4];130:13–22. Available from: https://doi.org/10.1016/j.jclinepi.2020.10.007

16. Tricco AC, Garritty CM, Boulos L, Lockwood C, Wilson M, McGowan J, et al. Rapid review methods more challenging during COVID-19: commentary with a focus on 8 knowledge synthesis steps [Internet]. Vol. 126, Journal of Clinical Epidemiology. Elsevier Inc.; 2020 [cited 2021 Oct 6]. p. 177–83. Available from: http://www.jclinepi.com/article/S0895435620306168/fulltext

17. Peters MDJ, Marnie C, Tricco AC, Pollock D, Munn Z, Alexander L, et al. Updated methodological guidance for the conduct of scoping reviews. JBI Evid Synth [Internet]. 2020 Oct 1 [cited 2021 Oct 6];18(10):2119–26. Available from: https://journals.lww.com/10.11124/JBIES-20-00167

18. Thomas J, Harden A. Methods for the thematic synthesis of qualitative research in systematic reviews. BMC Med Res Methodol [Internet]. 2008 Dec 10 [cited 2021 Oct 5];8(1):45. Available from: https://bmcmedresmethodol.biomedcentral.com/articles/10.1186/1471-2288-8-45

19. Levac D, Colquhoun H, O'Brien KK. Scoping studies: Advancing the methodology. Implement Sci [Internet]. 2010 Sep 20 [cited 2021 Oct 5];5(1):69. Available from: http://implementationscience.biomedcentral.com/articles/10.1186/1748-5908-5-69

20. Nowell LS, Norris JM, White DE, Moules NJ. Thematic Analysis: Striving to Meet the Trustworthiness Criteria. Int J Qual Methods [Internet]. 2017 Dec 2 [cited 2021 Oct 13];16(1):160940691773384. Available from: http://journals.sagepub.com/doi/10.1177/1609406917733847



For peer review only - http://bmjopen.bmj.com/site/about/guidelines.xhtml

BMJ Open

# Defining and identifying critical elements of, and lessons learned from addressing, 'operational readiness' for public health emergency events, including COVID-19: A rapid scoping review protocol

| | |
|---|---|
| Journal: | *BMJ Open* |
| Manuscript ID | bmjopen-2021-060526.R2 |
| Article Type: | Protocol |
| Date Submitted by the Author: | 11-Aug-2022 |
| Complete List of Authors: | English, Rene; Stellenbosch University Faculty of Medicine and Health Sciences, Global Health<br>Nyasulu, Juliet; Stellenbosch University, Faculty of Medicine and Health Sciences, Global Health Faculty of Medicine and Health Sciences Division of Health Systems and Public Health; AFRIQUIP, Health Systems Strengthening<br>Berner, Karina; Stellenbosch University Faculty of Medicine and Health Sciences, Rehabilitation Sciences<br>Geduld, Heike; Stellenbosch University, . Division of Emergency Medicine, Department of Family and Emergency Medicine, Faculty of Medicine and Health Sciences<br>McCaul, Michael; Stellenbosch University Faculty of Medicine and Health Sciences, Department of Interdisciplinary Sciences; Stellenbosch<br>Joseph, Conran; Stellenbosch University, Division of Physiotherapy, Department of Health and Rehabilitation Sciences,<br>Pappin, Michele; Stellenbosch University, Faculty of Medicine and Health Sciences, Global Health Faculty of Medicine and Health Sciences Division of Health Systems and Public Health<br>Gobat, Nina; World Health Organization<br>Boulanger, Linda; WHO<br>Louw, QA; Stellenbosch University, Physiotherapy; Stellenbosch University, |
| <b>Primary Subject Heading</b>: | Infectious diseases |
| Secondary Subject Heading: | Emergency medicine, Infectious diseases, Public health |
| Keywords: | INFECTIOUS DISEASES, PUBLIC HEALTH, ACCIDENT & EMERGENCY MEDICINE |
| | |

SCHOLARONE™
Manuscripts

# Defining and identifying critical elements of, and lessons learned from addressing, ‘operational readiness’ for public health emergency events, including COVID-19: a rapid scoping review protocol

**Word Count: Main document:** 3505 **Abstract :** 298

For peer review only



For peer review only - http://bmjopen.bmj.com/site/about/guidelines.xhtml

# Abstract

**Introduction:** Much is known around public health preparedness and response phases. However, between the two is phases is operational readiness which comprises the immediate actions needed to respond to a developing risk or hazard. Currently emergency readiness is embedded in multiple frameworks and policy documents related to the health emergency cycle. However, knowledge about operational readiness' critical readiness components and actions required by countries to respond to public health eminent threat is not well known. Therefore, we aim to define and identify the critical elements of 'operational readiness' for public health emergencies, including COVID-19, and identify lessons learnt from addressing it, to inform the World Health Organisation (WHO) Operational Readiness Framework.

**Methods and analysis:** This is a scoping review following the Joanna Briggs Institute guidance. Reporting will be according to the PRISMA Extension for Scoping Reviews (PRISMA-ScR) checklist. MEDLINE, Embase and Web of Science databases and grey literature will be searched and exported into an online systematic review software (e.g. Rayyan in this case) for review. The review team, which apart from scoping review methodological experts include content experts in health systems and public health and emergency medicine, prepared an a priori study protocol in consultation with WHO representatives. ATLAS.ti V9 will be used to conduct thematic data analysis as well as store, organise, and retrieve data. Data analysis and presentation will be carried out by five reviewers.

**Ethics and Dissemination:** This review will reveal new insights, knowledge and lessons learnt that will translate into an operational framework for readiness actions. In consultation with WHO, findings will be disseminated as appropriate (e.g., through professional bodies, conferences, and research papers). No ethics approvals are required as no humans will be involved in data collection.

**Protocol registration:** This rapid scoping review has been registered on Open Science Framework (doi:10.17605/OSF.IO/6SYAH).


For peer review only



For peer review only - http://bmjopen.bmj.com/site/about/guidelines.xhtml

## Study strengths and limitations

- The Covid-19 pandemic has shown that globally, countries even with well-resourced health systems and structured emergency preparedness plans in place were not able to sufficiently respond to the threat. Meaning that gaps existed between transition from preparedness to responding which is readiness. Therefore, defining and identifying critical elements of operational readiness for public health emergency events, including COVID-19 is critical
- Currently emergency operational readiness is embedded between preparedness and response and in most cases poorly defined. Therefore, we believe that an understanding of health systems readiness in responding to emergency is key.
- The review team included members that provided a mix of methodological and content expertise that will aid decisions regarding a speed-rigour trade-off.
- Currently there is no clear definition of activities that constitute health systems emergency readiness and people use different names whilst others name it either preparedness or response. In case these operational readiness definition words are not captured in the scoping review search strategy, this will be a limitation.
- Limiting the search to English full texts and last 11 years may lead to and publication timeline biases

For peer review only



For peer review only - http://bmjopen.bmj.com/site/about/guidelines.xhtml

# Introduction

Much has been documented about how countries should best prepare to respond to health emergencies (1–3). The effectiveness of 'readiness' – a concept referring to actions needed to rapidly respond to an imminently anticipated risk or hazard – largely depends on the sufficiency and comprehensiveness of prior longer-term 'preparedness' policies (4). However, little is known about the critical components of readiness and the kinds of readiness actions that should be taken by countries at all levels in response to health emergencies. Such knowledge is critical to inform operational readiness actions for future events.

Health Emergencies and Disaster Risk Management (Health-EDRM) encompasses the intersecting fields of emergency and disaster medicine, health systems strengthening and resilience, disaster risk reduction, humanitarian response and community health resilience. Within this framework, it is accepted that the management of emergencies is a whole-of-society approach, focusing on all hazards and involving multiple sectors and multiple disciplines (5). Health-EDRM involves four broad components, namely (i) hazard vulnerability assessment (HVA) and mitigation; (ii) preparedness; (iii) response and (iv) recovery. Within these, the activities of 'readiness' will occur within both HVA and mitigation and preparedness components. These readiness activities are linked both temporally and structurally to a specifically identified hazard, whether that is an infectious disease, or climate change event. Thus, what constitutes 'readiness' is determined by the nature of the hazard.

The World Health Organisation (WHO) Strategic Framework for Emergency Preparedness (6) is a unifying framework for country-level public health emergency preparedness. This framework describes operational readiness to respond to emergencies as a continuous, co-ordinated process, involving a multisectoral response, incorporating multiple level infrastructure, and following an all-hazard approach with a focus on high priority risks (6).

The current COVID-19 global pandemic has exposed the fragility of health systems to respond to shocks in the form of disease outbreaks or health emergencies (7). According to the WHO, the response of a public health system to an outbreak or health emergency such as the COVID-19 pandemic can be defined as a cycle that sways between preparedness and the actual response. Through applying a governance lens, the WHO has developed an Emergency Response Framework (4), which describes the stages of an outbreak or health emergency. As alluded to above, readiness to respond lies somewhere between preparedness and response; it is the instant action to an emergent or prominent risk and is hugely reliant on adequate preparedness (4). In many instances, implementation of these well-designed disaster preparedness policies is met with significant challenges due to flaws in the 'readiness' of systems to do so. 'Readiness' as a concept has not been fully designed, and therefore it is critical to define the critical components of readiness and the types of readiness actions to be taken in response to outbreaks and health emergencies to inform operational readiness actions for future events (8). A preliminary search of MEDLINE, the Cochrane Database of



For peer review only - http://bmjopen.bmj.com/site/about/guidelines.xhtml

Systematic Reviews, Prospero and JBI Evidence Synthesis revealed no current or underway systematic reviews or scoping reviews on the topic. The WHO is currently developing an Operational Readiness Framework intended to guide effective action. Specifically, the purpose of the framework is to scale-up preparedness for a specific risk at the local and national levels by considering how ready a country is to respond to the imminent threat, and to identify key actions needed to be ready to respond effectively to that threat. To this end, WHO has called for a rapid scoping review to be conducted that will assist with defining available evidence related to readiness and readiness actions.

# Aim and objectives

The overarching aim of this rapid scoping review is to define and identify the critical elements of 'operational readiness' for public health emergencies, including COVID-19, and identify lessons learnt from addressing it, to inform the WHO Operational Readiness Framework.

To this end, the following objectives will be addressed:

1. To conceptualise and define 'operational readiness'.
2. To map and describe frameworks, policies and evidence/information related to 'operational readiness' for all hazards, with a strong focus on infectious diseases.
3. To define critical elements of 'operational readiness' at multiple levels of the health system (community, local, sub-national, national, regional, global).
4. To identify lessons learned from enhancing or influencing 'operational readiness' (at multiple levels).

# Review question

## Primary scoping review method question

The primary review question was formulated using the PCC (Population, Concept and Context) method (9): *How can/do communities/countries/regions/global institutions operationalise readiness for imminent public emergencies?*

## Sub-questions

The review will seek to answer the following additional or sub-questions:

1. How is 'operational readiness' for public health emergencies conceptualised and defined?
2. What are the critical elements (dimensions, operational actions, coordination) of 'operational readiness' for public health emergencies at multiple levels (community, local, sub-national, national, regional, global)?
3. How did countries ready/ prepare for COVID-19?



For peer review only - http://bmjopen.bmj.com/site/about/guidelines.xhtml

4. What lessons have been learned about 'operational readiness' during for example, COVID-19/ Ebola, with a strong focus on infectious disease emergencies?

# Keywords

All hazards; Disaster planning; Epidemic; Imminent threat; Infectious diseases; Outbreak; Pandemic; Public health emergency

# Eligibility criteria

## Inclusion criteria

### *Participants/ population*

These are the groups or organisations who would respond and/or lead the response, and include the following:

- Communities (local, subregional, or national level)
- National, country, regional and global governments
- Global health organisations, such as the WHO

### *Concept*

The purpose of the scoping review is to define 'operational readiness. This concept refers to the immediate action(s) that are taken to pre-position response actions needed to address a proximal, imminent hazard/ threat – such as an 'acute' infectious disease outbreak or natural disaster threat (an all-hazards approach). These include but not limited to Disease Outbreaks, epidemics/ or pandemics, public health emergency, communicable diseases, Incident Management System, country risk profile and many other details. The concept lies between 'preparedness' and 'response'. To find evidence of readiness interventions, we will look at sources referencing preparedness, planning and disaster management as the term 'readiness' may be embedded in 'preparedness' – or the term 'preparedness' may be used to describe actions that (based on our definition) we would describe as readiness.

We will consider sources that:

- Conceptualise, theorise, define, or describe or interpret 'operational readiness' and/or preparedness for public health emergencies (at community, country, regional or global levels) at the time when the threat of an infectious disease outbreaks or natural disaster becomes known, within a specific timeframe (viz., defining 'imminence').



For peer review only - http://bmjopen.bmj.com/site/about/guidelines.xhtml

- Contain explanations, descriptions, intervention approaches, analysis or frameworks or anticipatory actions for 'operational readiness' or preparedness for public health emergencies (at community, country, regional or global levels) when the threat of an infectious disease outbreaks or natural disaster becomes known.
- Provide the nature and description of critical elements (dimensions, coordination, roles of key stakeholders such as the community, health actors, policy makers etc.) of 'operational readiness' for public health emergencies at community, national, regional, and global levels.

### *Context*

The context of health emergencies refers to natural disasters and infectious disease threats (new and re-emerging) – i.e., all hazards. Important to note is that these threats are acute (imminent) and impact the health of populations. These health emergencies occur within the community as well as health system and health service contexts.

The proposed definition of a 'health emergency' is an extraordinary event that is determined to 'constitute a public health risk whose scale, timing, or unpredictability threatens to overwhelm routine capabilities of the health system' (10 pS9) and potentially require a coordinated response at multiple levels (10,11).

### *Types of sources*

- Peer-reviewed review or empirical research (any study design) that is available in full-text and published in scientific journals between 2010 and 2021.
- Publicly available policy frameworks and programme reports.
  Published conference reports or electronic theses.
- Documents of which the full text or abstract is available in the English language. If the English version of the abstract is potentially eligible for inclusion, the full text (if German/ French/ Afrikaans) will be translated to make a final decision on eligibility.

### Exclusion criteria

- Papers focusing exclusively on longer term preparedness actions or exclusively on response actions will be excluded.
- Papers reporting on contexts beyond health emergencies or not focused on disease prevention and control will be excluded.

## Methods and data analysis

This rapid scoping review will be conducted in accordance with the Joanna Briggs Institute (JBI) methodology for scoping reviews (9). The review will be reported using the PRISMA Extension for Scoping Reviews (PRISMA-ScR) (12) and PRISMA-S Extension for Searches in Systematic Reviews (13).



For peer review only - http://bmjopen.bmj.com/site/about/guidelines.xhtml

## Search strategy

The search strategy will aim to locate peer-reviewed review or empirical research (any study design) that is available in full-text and published in scientific journals, publicly available policy frameworks, programme reports, and published conference reports or electronic theses. This will include humanitarian literature where health impacts or effects are the focus. Due to the rapid nature of the scoping review, we will restrict the search to studies published between 2010 – 2021 and those available in English (potentially eligible Afrikaans, German or French full texts, according to the English abstract, will be translated into English).

The electronic databases to be searched include MEDLINE, Embase and Web of Science. An initial limited search of MEDLINE was undertaken to identify articles on the review topic. The text words contained in the titles and abstracts of relevant articles, and the index terms used to describe the articles were used to draft a full search strategy for MEDLINE. The search strings and terms were developed iteratively and in consultation with the WHO and are centred around three key concepts: (i) emergencies/ diseases/ natural disasters; (ii) readiness/ preparedness/ risk/ planning; and (iii) health systems/ community. The search strategy, including all identified keywords and index terms, was subsequently adapted for Embase and Web of Science. Searches will be conducted by an expert information specialist in consultation with the review team. The reference list of all included sources of evidence will also be screened for additional studies. Reporting of the searching will be guided by the PRISMA-S Extension for Searches in Systematic Reviews (13).

### *Searching other resources*

Sources of unpublished studies/ grey literature to be searched include various targeted repositories, websites, and databases. These include global organisations (e.g., the WHO, United Nation Children's Fund [UNICEF], United Nations Office for Disaster Risk Reduction [UNDRR], United Nations International Strategy for Disaster Reduction [UNISDR], International Federation of Red Cross [IFRC], International Committee of the Red Cross [ICRC]), regional WHO offices (i.e., Southeast Asian, African, Western Pacific, Pan American, European and Eastern Mediterranean) and the European Centre for Disaster Medicine (CEMEC). Societies and organisations include the World Association for Disaster and Emergency Medicine (WADEM), Médecins Sans Frontières (MSF) and ReliefWeb. National websites include the United States Centres for Disease Control and Prevention (CDC) and Federal Emergency Management Agency (FEMA), the Robert Koch Institute (RKI), Public Health England. Lastly, Evidence Aid will be included as an evidence repository.

## Selection of studies

All search hits will be imported into Rayyan V0.1.0 software (Rayyan Systems Inc., MA, USA) (14) for screening, checking of duplicates and selection of final documents to be included. To support consistent abstract and title screening and refine eligibility, senior authors (RE, HG and



For peer review only - http://bmjopen.bmj.com/site/about/guidelines.xhtml

MM) together with the title and abstract screeners (MP and MYC), will (as an initial step) independently and in duplicate screen 100 articles, followed by discussion. The following proposed screening approach is adapted from the Cochrane Rapid Reviews Methods Group guidance for systematic reviews to balance rigour and speed consistent with rapid reviews (15,16). Twenty percent of titles and abstracts will be screened by two reviewers (MP and MYC), independently, in duplicate and with conflict resolution, to remove obviously irrelevant reports. After this, one reviewer (MP) will screen the remaining titles and abstracts while the second reviewer (MYC) will verify excluded titles and abstracts and resolve conflicts (15). If required, a third senior reviewer (HG or RE) will resolve any disagreements The full texts of selected citations will subsequently be assessed in detail against the eligibility criteria by the first reviewer, while the second reviewer will verify all excluded full texts (15). Reasons for exclusion of sources of evidence at full text that do not meet the inclusion criteria will be recorded. This information will be reported and added to a table of excluded studies in the scoping review. Any disagreements that arise between the reviewers at each stage of the selection process will be resolved through discussion, including with an additional senior reviewer (HG or RE) if needed. If study eligibility is unclear owing to missing data, further information will be requested from study authors. The results of the search and the source inclusion process will be reported in full in the final scoping review and presented in a PRISMA-ScR flow diagram (12).

## Data extraction and management

Due to the rapid design and potential large pool of included studies we will use a dynamic approach to data extraction and management. For an included study yield of ≤25, data extraction will be done by one reviewer (MP), while a second reviewer (KB or MYC) will check for completeness and accuracy (15). For yields between >25 but ≤75, two or more extractors will be used (e.g., MP/KB/MYC/CJ/QL/RE), while an additional reviewer will check for correctness and accuracy (17). In the case of more than 75 included sources, we will consider a prioritisation process whereby we rank or stratify studies based on design and relevancy to the scoping review. Prioritised studies will then be included for data extraction until the review team, together with WHO, agrees that data saturation has been achieved. The reviewers will discuss the nature of the information that will be extracted before commencing the process to facilitate coherence. Any uncertainties before and during the extraction process will be discussed with team members to make a final decision.

The data extracted will include author name(s), publication year, publication country and World Bank classification, source classification as primary/ secondary/ multi-method, publication type, study design, aim/ purpose, sample/ facility description, method/ tool for data collection, modifications to the data tool (if any), level (community, national, etc.), type of emergency, operational readiness definition, preparedness definition, key actors, challenges/ recommendations, lessons learnt, and other relevant information/ conclusions. In addition,



For peer review only - http://bmjopen.bmj.com/site/about/guidelines.xhtml

data regarding readiness will be extracted according to the WHO's operational readiness components – these include:

- Leadership, governance, and coordination,
- Country risk profile,
- Operational planning and coordination,
- Contingency finance,
- Health facility capacity and service delivery,
- Health workforce/ human resources,
- Early warning or surveillance and health information systems,
- Community resilience and risk communications,
- Logistics or supply chain for access to essential medicines,
- WHO readiness,
- Partner readiness.

Framework details and any associated actions will be recorded. Finally, information regarding relevant models will be extracted, including URL links to figures/ diagrams.

A draft extraction form will be pilot-tested independently by two reviewers using a sample of two to three potential included full-text articles/ evidence sources (17). Based on feedback from the two reviewers, the form may be modified and revised as necessary during the process of extracting data from each included evidence source (17). Necessary modifications will first be discussed within the review team for consensus, and any changes implemented will be reported in the final scoping review. Authors will be contacted where possible to clarify or obtain additional information.

## Methodological appraisal

Included peer-reviewed literature will be evaluated for quality based on appropriate pre-existing methodological quality checklists.

## Data analysis and presentation

Data will be synthesised in line with the core objectives of the rapid scoping review.

The included documents will be analysed using qualitative thematic analysis through an deductive synthesis approach (18–20). We are proposing to use ATLAS.ti V8 (Scientific Software Development GmbH) (https://atlasti.com/) to conduct thematic data analysis as well as store, organise, and retrieve data. Data analysis will be carried out by the project group researchers, who have vast knowledge and experience in undertaking reviews, including scoping reviews, that have used qualitative thematic analysis.

Findings will be deductively coded into a conceptual model that is taken from the WHO Country Readiness for Health Checklist to define and identify the critical elements of



For peer review only - http://bmjopen.bmj.com/site/about/guidelines.xhtml

'operational readiness' for public health emergencies, including COVID-19, and identify lessons learnt from addressing it. We will also identify if there are additional consistent themes emerging from the analysis that are not currently included in the WHO Checklist, as potential additional items.

The analysis will start by evaluating documented text line-by-line, allocating text a descriptive label and code. The same will be done for the other focused questions on understanding the similarities and differences between operational readiness and preparedness and identifying critical elements. The researchers will remain close to the data from the primary sources when defining and understanding the meaning structure of these concepts and phenomena. Since the conceptual understanding of 'operational readiness' and 'preparedness' will be initially explored, described, and theorised and may vary across sources, we will initially use broad, higher order codes (which may form main themes) developed deductively from the framework to organise the data. Once all data have been initially coded and collated, all the potentially relevant coded data extracts will be sorted and collated into themes and sub-themes (including a 'miscellaneous' theme for codes that do not clearly fit into existing themes (20). Senior reviewers (RE, HG and QL) will debrief the researchers primarily responsible for the thematic analysis, and the review team will meet regularly to discuss codes and themes, including potential merging or further break-down of themes (depending on whether there are enough data to support a theme, or the data are deemed too diverse). The themes will represent the synthesis and interpretation that go beyond the primary sources as well as deliver new insights and knowledge, which will translate into an operational framework for readiness and important lessons learnt.

A numerical description of the extent and nature of included evidence sources will be presented using tables and charts, accompanied by narrative summaries to describe how the results relate to the review's objectives.

## Patient Public involvement

Patients or the public were not involved in the design, or conduct, or reporting, or dissemination plans of our research

## Ethics, reporting and dissemination

No ethical approval is needed for this rapid scoping review, given that included sources will comprise of published and publicly available information.

The study was expected to commerce in December 2021 to July 2022 with first scientific publication output expected in August 2022. The Stellenbosch University (SU) review team will work with the WHO commissioning group and draw on the expertise of expert advisors to the review team to produce the following outputs. Weekly internal and SU-WHO meetings have been conducted to provide input into the development of this research protocol and will



For peer review only - http://bmjopen.bmj.com/site/about/guidelines.xhtml

continue to aid understanding of emerging insights and findings that can inform work tasks relevant to the technical product development. Interim findings from the rapid scoping review will be presented to the WHO. Following feedback, an updated interim report incorporating feedback from the WHO and expert advisory team will be presented. The final report of the full rapid scoping review will be delivered, along with a PowerPoint presentation to the WHO commissioning group of findings with talking points. In consultation with the WHO, findings will be disseminated further as appropriate (e.g., through professional bodies, conferences, and research papers). By defining evidence related to critical readiness components and actions, this review will reveal new insights, knowledge and lessons learnt that will translate into an operational framework for readiness actions.


## Acknowledgements

The rapid scoping review was commissioned by the WHO to inform an Operational Readiness Framework for the Country Readiness Strengthening Department in the World Health Emergencies Program in WHO (Reference #: 2021/1145765; Unit: MST; Cluster: QNF/SCI).


## Authors' contribution

RE is the Principal investigator and JCYN coordinates the team, the research process and is a corresponding author. NG and LB contributed to content through the process, provided guidance on the expected outcomes and WHO guidelines to be followed. KB and MP drafted the original protocol, MM, QL, CJ and HG provided technical guidance on the methodology. All authors were involved in the conceptualization of the concept paper and proposal writing and read the final version of the submitted protocol.


## Funding

This work is supported by the World Health Organization through a consultancy fee and not research grant [Reference: APW/RR/Readiness/2021/1145765], as a result, there is no grant number


## Conflicts of interest

There is no conflict of interest in this project.



For peer review only - http://bmjopen.bmj.com/site/about/guidelines.xhtml

For peer review only

## References


1. Savoia E, Lin L, Viswanath K. Communications in public health emergency preparedness: A systematic review of the literature [Internet]. Vol. 11, Biosecurity and Bioterrorism. Mary Ann Liebert, Inc.; 2013 [cited 2021 Oct 4]. p. 170–84. Available from: /pmc/articles/PMC3778998/

2. Tang R, Fitzgerald G, Hou XY, Wu YP. Building an evaluation instrument for china's hospital emergency preparedness: A systematic review of preparedness instruments [Internet]. Vol. 8, Disaster Medicine and Public Health Preparedness. Lippincott Williams and Wilkins; 2014 [cited 2021 Oct 4]. p. 101–9. Available from: https://pubmed.ncbi.nlm.nih.gov/24622400/

3. Ejeta LT, Ardalan A, Paton D. Application of behavioral theories to disaster and emergency health preparedness: A systematic review. PLoS Curr. 2015 Jul;7(DISASTERS).

4. WHO Emergencies Preparedness Team. Emergency response framework [Internet]. 2nd ed. World Health Organization, editor. Geneva; 2017. Available from: https://www.who.int/publications/i/item/9789241512299

5. Lo STT, Chan EYY, Chan GKW, Murray V, Abrahams J, Ardalan A, et al. Health Emergency and Disaster Risk Management (Health-EDRM): Developing the Research Field within the Sendai Framework Paradigm. Int J Disaster Risk Sci [Internet]. 2017 Jun 1 [cited 2021 Oct 4];8(2):145–9. Available from: www.ijdrs.comwww.springer.com/13753

6. WHO Emergencies Preparedness Team. A strategic framework for emergency preparedness [Internet]. World Health Organization, editor. Geneva; 2016. Available from: https://www.who.int/publications/i/item/a-strategic-framework-for-emergency-preparedness

7. Haldane V, De Foo C, Abdalla SM, Jung AS, Tan M, Wu S, et al. Health systems resilience in managing the COVID-19 pandemic: lessons from 28 countries [Internet]. Vol. 27, Nature Medicine. Nature Research; 2021 [cited 2021 Oct 4]. p. 964–80. Available from: https://pubmed.ncbi.nlm.nih.gov/34002090/

8. Khan Y, O'Sullivan T, Brown A, Tracey S, Gibson J, Généreux M, et al. Public health emergency preparedness: A framework to promote resilience. BMC Public Health [Internet]. 2018 Dec 5 [cited 2021 Oct 4];18:1344. Available from: https://bmcpublichealth.biomedcentral.com/articles/10.1186/s12889-018-6250-7

9. Peters M, Godfrey C, McInerey P, Munn Z, Tricco A, Khalil H. Chapter 11: Scoping Reviews (2020 version). In: Aromataris E, Munn Z, editors. JBI Manual for Evidence Synthesis [Internet]. JBI; 2020. Available from: https://synthesismanual.jbi.global

10. Nelson C, Lurie N, Wasserman J, Zakowski S. Conceptualizing and defining public health emergency preparedness. Vol. 97 Suppl 1, American journal of public health. American Public Health Association; 2007. p. S9–11.

11. World Health Organization. Chapter 11: Public health emergencies. In: Advancing the




For peer review only - http://bmjopen.bmj.com/site/about/guidelines.xhtml

right to health: the vital role of law [Internet]. Geneva: World Health Organization; 2017. Available from: https://apps.who.int/iris/handle/10665/252815

12. Tricco AC, Lillie E, Zarin W, O'Brien KK, Colquhoun H, Levac D, et al. PRISMA Extension for Scoping Reviews (PRISMA-ScR): Checklist and Explanation. Ann Intern Med [Internet]. 2018 Oct 2 [cited 2019 Apr 8];169(7):467–74. Available from: http://annals.org/article.aspx?doi=10.7326/M18-0850

13. Rethlefsen ML, Kirtley S, Waffenschmidt S, Ayala AP, Moher D, Page MJ, et al. PRISMA-S: an extension to the PRISMA Statement for Reporting Literature Searches in Systematic Reviews. Syst Rev [Internet]. 2021 Jan 26 [cited 2021 Oct 4];10(1):39. Available from: https://doi.org/10.1186/s13643-020-01542-z

14. Ouzzani M, Hammady H, Fedorowicz Z, Elmagarmid A. Rayyan -a web and mobile app for systematic reviews. Syst Rev [Internet]. 2016 [cited 2021 Oct 4];5(1):210. Available from: http://systematicreviewsjournal.biomedcentral.com/articles/10.1186/s13643-016-0384-4

15. Garritty C, Gartlehner G, Nussbaumer-Streit B, King VJ, Hamel C, Kamel C, et al. Cochrane Rapid Reviews Methods Group offers evidence-informed guidance to conduct rapid reviews. J Clin Epidemiol [Internet]. 2021 Feb 1 [cited 2021 Oct 4];130:13–22. Available from: https://doi.org/10.1016/j.jclinepi.2020.10.007

16. Tricco AC, Garritty CM, Boulos L, Lockwood C, Wilson M, McGowan J, et al. Rapid review methods more challenging during COVID-19: commentary with a focus on 8 knowledge synthesis steps [Internet]. Vol. 126, Journal of Clinical Epidemiology. Elsevier Inc.; 2020 [cited 2021 Oct 6]. p. 177–83. Available from: http://www.jclinepi.com/article/S0895435620306168/fulltext

17. Peters MDJ, Marnie C, Tricco AC, Pollock D, Munn Z, Alexander L, et al. Updated methodological guidance for the conduct of scoping reviews. JBI Evid Synth [Internet]. 2020 Oct 1 [cited 2021 Oct 6];18(10):2119–26. Available from: https://journals.lww.com/10.11124/JBIES-20-00167

18. Thomas J, Harden A. Methods for the thematic synthesis of qualitative research in systematic reviews. BMC Med Res Methodol [Internet]. 2008 Dec 10 [cited 2021 Oct 5];8(1):45. Available from: https://bmcmedresmethodol.biomedcentral.com/articles/10.1186/1471-2288-8-45

19. Levac D, Colquhoun H, O'Brien KK. Scoping studies: Advancing the methodology. Implement Sci [Internet]. 2010 Sep 20 [cited 2021 Oct 5];5(1):69. Available from: http://implementationscience.biomedcentral.com/articles/10.1186/1748-5908-5-69

20. Nowell LS, Norris JM, White DE, Moules NJ. Thematic Analysis: Striving to Meet the Trustworthiness Criteria. Int J Qual Methods [Internet]. 2017 Dec 2 [cited 2021 Oct 13];16(1):160940691773384. Available from: http://journals.sagepub.com/doi/10.1177/1609406917733847

For peer review only



For peer review only - http://bmjopen.bmj.com/site/about/guidelines.xhtml

BMJ Open

# Defining and identifying critical elements of, and lessons learned from addressing, ‘operational readiness’ for public health emergency events, including COVID-19: A rapid scoping review protocol

| | |
|---:|---|
| Journal: | *BMJ Open* |
| Manuscript ID | bmjopen-2021-060526.R3 |
| Article Type: | Protocol |
| Date Submitted by the Author: | 22-Aug-2022 |
| Complete List of Authors: | English, Rene; Stellenbosch University Faculty of Medicine and Health Sciences, Global Health<br>Nyasulu, Juliet; Stellenbosch University, Faculty of Medicine and Health Sciences, Global Health Faculty of Medicine and Health Sciences Division of Health Systems and Public Health; AFRIQUIP, Health Systems Strengthening<br>Berner, Karina; Stellenbosch University Faculty of Medicine and Health Sciences, Rehabilitation Sciences<br>Geduld, Heike; Stellenbosch University, . Division of Emergency Medicine, Department of Family and Emergency Medicine, Faculty of Medicine and Health Sciences<br>McCaul, Michael; Stellenbosch University Faculty of Medicine and Health Sciences, Department of Interdisciplinary Sciences; Stellenbosch<br>Joseph, Conran; Stellenbosch University, Division of Physiotherapy, Department of Health and Rehabilitation Sciences,<br>Pappin, Michele; Stellenbosch University, Faculty of Medicine and Health Sciences, Global Health Faculty of Medicine and Health Sciences Division of Health Systems and Public Health<br>Gobat, Nina; World Health Organization<br>Boulanger, Linda; WHO<br>Louw, QA; Stellenbosch University, Physiotherapy; Stellenbosch University, |
| <b>Primary Subject Heading</b>: | Infectious diseases |
| Secondary Subject Heading: | Emergency medicine, Infectious diseases, Public health |
| Keywords: | INFECTIOUS DISEASES, PUBLIC HEALTH, ACCIDENT & EMERGENCY MEDICINE |
| | |

SCHOLARONE™
Manuscripts

For peer review only - http://bmjopen.bmj.com/site/about/guidelines.xhtml

# Defining and identifying critical elements of, and lessons learned from addressing, 'operational readiness' for public health emergency events, including COVID-19: A scoping review protocol


## Authors

René English[1 *] Juliet Charity Yauka Nyasulu[1,2] Karina Berner[3] Heike Geduld[4] Michael McCaul[5] Conran Joseph[3] Michele Pappin[1] Nina Gobat[6], Lucy Boulanger[–6] Quinette Louw[3]

1. Division of Health Systems and Public Health, Department of Global Health, Faculty of Medicine and Health Sciences, Stellenbosch University, Cape Town 8000, South Africa
2. AFRIQUIP, Health Systems Strengthening Johannesburg, ZA
3. Division of Physiotherapy, Department of Health and Rehabilitation Sciences, Faculty of Medicine and Health Sciences, Stellenbosch University, Cape Town 8000, South Africa
4. Division of Emergency Medicine, Department of Family and Emergency Medicine, Faculty of Medicine and Health Sciences, Stellenbosch University, Cape Town 8000, South Africa
5. Centre for Evidence-based Health Care, Division of Epidemiology and Biostatistics, Department of Global Health, Stellenbosch University 8000, South Africa
6. Community Readiness and Resilience Unit, Country Readiness Strengthening, WHO World Health Emergencies Program, Geneva, Switzerland

**Corresponding author: Dr Juliet C Y Nyasulu,** jnyasulu@sun.ac.za, Division of Health Systems and Public Health, Department of Global Health, Faculty of Medicine and Health Sciences, Stellenbosch University, Cape Town 8000, South Africa


**Word Count: Main document:** 3505 **Abstract :** 298

For peer review only

# Abstract

**Introduction:** Much is known around public health preparedness and response phases. However, between the two is phases is operational readiness which comprises the immediate actions needed to respond to a developing risk or hazard. Currently emergency readiness is embedded in multiple frameworks and policy documents related to the health emergency cycle. However, knowledge about operational readiness' critical readiness components and actions required by countries to respond to public health eminent threat is not well known. Therefore, we aim to define and identify the critical elements of 'operational readiness' for public health emergencies, including COVID-19, and identify lessons learnt from addressing it, to inform the World Health Organisation (WHO) Operational Readiness Framework.

**Methods and analysis:** This is a scoping review following the Joanna Briggs Institute guidance. Reporting will be according to the PRISMA Extension for Scoping Reviews (PRISMA-ScR) checklist. MEDLINE, Embase and Web of Science databases and grey literature will be searched and exported into an online systematic review software (e.g. Rayyan in this case) for review. The review team, which apart from scoping review methodological experts include content experts in health systems and public health and emergency medicine, prepared an a priori study protocol in consultation with WHO representatives. ATLAS.ti V9 will be used to conduct thematic data analysis as well as store, organise, and retrieve data. Data analysis and presentation will be carried out by five reviewers.

**Ethics and Dissemination:** This review will reveal new insights, knowledge and lessons learnt that will translate into an operational framework for readiness actions. In consultation with WHO, findings will be disseminated as appropriate (e.g., through professional bodies, conferences, and research papers). No ethics approvals are required as no humans will be involved in data collection.

**Protocol registration:** This rapid scoping review has been registered on Open Science Framework (doi:10.17605/OSF.IO/6SYAH).


For peer review only



For peer review only - http://bmjopen.bmj.com/site/about/guidelines.xhtml

## Study strengths and limitations

- The Covid-19 pandemic has shown that globally, countries even with well-resourced health systems and structured emergency preparedness plans in place were not able to sufficiently respond to the threat. Meaning that gaps existed between transition from preparedness to responding which is readiness. Therefore, defining and identifying critical elements of operational readiness for public health emergency events, including COVID-19 is critical
- Currently emergency operational readiness is embedded between preparedness and response and in most cases poorly defined. Therefore, we believe that an understanding of health systems readiness in responding to emergency is key.
- The review team included members that provided a mix of methodological and content expertise that will aid decisions regarding a speed-rigour trade-off.
- Currently there is no clear definition of activities that constitute health systems emergency readiness and people use different names whilst others name it either preparedness or response. In case these operational readiness definition words are not captured in the scoping review search strategy, this will be a limitation.
- Limiting the search to English full texts and last 11 years may lead to and publication timeline biases



For peer review only - http://bmjopen.bmj.com/site/about/guidelines.xhtml

# Introduction

Much has been documented about how countries should best prepare to respond to health emergencies (1–3). The effectiveness of 'readiness' – a concept referring to actions needed to rapidly respond to an imminently anticipated risk or hazard – largely depends on the sufficiency and comprehensiveness of prior longer-term 'preparedness' policies (4). However, little is known about the critical components of readiness and the kinds of readiness actions that should be taken by countries at all levels in response to health emergencies. Such knowledge is critical to inform operational readiness actions for future events.

Health Emergencies and Disaster Risk Management (Health-EDRM) encompasses the intersecting fields of emergency and disaster medicine, health systems strengthening and resilience, disaster risk reduction, humanitarian response and community health resilience. Within this framework, it is accepted that the management of emergencies is a whole-of-society approach, focusing on all hazards and involving multiple sectors and multiple disciplines (5). Health-EDRM involves four broad components, namely (i) hazard vulnerability assessment (HVA) and mitigation; (ii) preparedness; (iii) response and (iv) recovery. Within these, the activities of 'readiness' will occur within both HVA and mitigation and preparedness components. These readiness activities are linked both temporally and structurally to a specifically identified hazard, whether that is an infectious disease, or climate change event. Thus, what constitutes 'readiness' is determined by the nature of the hazard.

The World Health Organisation (WHO) Strategic Framework for Emergency Preparedness (6) is a unifying framework for country-level public health emergency preparedness. This framework describes operational readiness to respond to emergencies as a continuous, co-ordinated process, involving a multisectoral response, incorporating multiple level infrastructure, and following an all-hazard approach with a focus on high priority risks (6).

The current COVID-19 global pandemic has exposed the fragility of health systems to respond to shocks in the form of disease outbreaks or health emergencies (7). According to the WHO, the response of a public health system to an outbreak or health emergency such as the COVID-19 pandemic can be defined as a cycle that sways between preparedness and the actual response. Through applying a governance lens, the WHO has developed an Emergency Response Framework (4), which describes the stages of an outbreak or health emergency. As alluded to above, readiness to respond lies somewhere between preparedness and response; it is the instant action to an emergent or prominent risk and is hugely reliant on adequate preparedness (4). In many instances, implementation of these well-designed disaster preparedness policies is met with significant challenges due to flaws in the 'readiness' of systems to do so. 'Readiness' as a concept has not been fully designed, and therefore it is critical to define the critical components of readiness and the types of readiness actions to be taken in response to outbreaks and health emergencies to inform operational readiness actions for future events (8). A preliminary search of MEDLINE, the Cochrane Database of Systematic Reviews, Prospero and JBI Evidence Synthesis revealed no



For peer review only - http://bmjopen.bmj.com/site/about/guidelines.xhtml

current or underway systematic reviews or scoping reviews on the topic. The WHO is currently developing an Operational Readiness Framework intended to guide effective action. Specifically, the purpose of the framework is to scale-up preparedness for a specific risk at the local and national levels by considering how ready a country is to respond to the imminent threat, and to identify key actions needed to be ready to respond effectively to that threat. To this end, WHO has called for a rapid scoping review to be conducted that will assist with defining available evidence related to readiness and readiness actions.

## Aim and objectives

The overarching aim of this rapid scoping review is to define and identify the critical elements of 'operational readiness' for public health emergencies, including COVID-19, and identify lessons learnt from addressing it, to inform the WHO Operational Readiness Framework.

To this end, the following objectives will be addressed:

1. To conceptualise and define 'operational readiness'.
2. To map and describe frameworks, policies and evidence/information related to 'operational readiness' for all hazards, with a strong focus on infectious diseases.
3. To define critical elements of 'operational readiness' at multiple levels of the health system (community, local, sub-national, national, regional, global).
4. To identify lessons learned from enhancing or influencing 'operational readiness' (at multiple levels).

## Review question

### Primary scoping review method question

The primary review question was formulated using the PCC (Population, Concept and Context) method (9): *How can/do communities/countries/regions/global institutions operationalise readiness for imminent public emergencies?*

### Sub-questions

The review will seek to answer the following additional or sub-questions:

1. How is 'operational readiness' for public health emergencies conceptualised and defined?
2. What are the critical elements (dimensions, operational actions, coordination) of 'operational readiness' for public health emergencies at multiple levels (community, local, sub-national, national, regional, global)?
3. How did countries ready/ prepare for COVID-19?
4. What lessons have been learned about 'operational readiness' during for example, COVID-19/ Ebola, with a strong focus on infectious disease emergencies?



For peer review only - http://bmjopen.bmj.com/site/about/guidelines.xhtml

# Keywords

All hazards; Disaster planning; Epidemic; Imminent threat; Infectious diseases; Outbreak; Pandemic; Public health emergency

# Eligibility criteria

## Inclusion criteria

### *Participants/ population*

These are the groups or organisations who would respond and/or lead the response, and include the following:

- Communities (local, subregional, or national level)
- National, country, regional and global governments
- Global health organisations, such as the WHO

### *Concept*

The purpose of the scoping review is to define 'operational readiness. This concept refers to the immediate action(s) that are taken to pre-position response actions needed to address a proximal, imminent hazard/ threat – such as an 'acute' infectious disease outbreak or natural disaster threat (an all-hazards approach). These include but not limited to Disease Outbreaks, epidemics/ or pandemics, public health emergency, communicable diseases, Incident Management System, country risk profile and many other details. The concept lies between 'preparedness' and 'response'. To find evidence of readiness interventions, we will look at sources referencing preparedness, planning and disaster management as the term 'readiness' may be embedded in 'preparedness' – or the term 'preparedness' may be used to describe actions that (based on our definition) we would describe as readiness.

We will consider sources that:

- Conceptualise, theorise, define, or describe or interpret 'operational readiness' and/or preparedness for public health emergencies (at community, country, regional or global levels) at the time when the threat of an infectious disease outbreaks or natural disaster becomes known, within a specific timeframe (viz., defining 'imminence').
- Contain explanations, descriptions, intervention approaches, analysis or frameworks or anticipatory actions for 'operational readiness' or preparedness for public health emergencies (at community, country, regional or global levels) when the threat of an infectious disease outbreaks or natural disaster becomes known.

For peer review only



For peer review only - http://bmjopen.bmj.com/site/about/guidelines.xhtml

- Provide the nature and description of critical elements (dimensions, coordination, roles of key stakeholders such as the community, health actors, policy makers etc.) of 'operational readiness' for public health emergencies at community, national, regional, and global levels.

### *Context*

The context of health emergencies refers to natural disasters and infectious disease threats (new and re-emerging) – i.e., all hazards. Important to note is that these threats are acute (imminent) and impact the health of populations. These health emergencies occur within the community as well as health system and health service contexts.

The proposed definition of a 'health emergency' is an extraordinary event that is determined to 'constitute a public health risk whose scale, timing, or unpredictability threatens to overwhelm routine capabilities of the health system' (10 pS9) and potentially require a coordinated response at multiple levels (10,11).

### *Types of sources*

- Peer-reviewed review or empirical research (any study design) that is available in full-text and published in scientific journals between 2010 and 2021.
- Publicly available policy frameworks and programme reports.
  Published conference reports or electronic theses.
- Documents of which the full text or abstract is available in the English language. If the English version of the abstract is potentially eligible for inclusion, the full text (if German/ French/ Afrikaans) will be translated to make a final decision on eligibility.

### Exclusion criteria

- Papers focusing exclusively on longer term preparedness actions or exclusively on response actions will be excluded.
- Papers reporting on contexts beyond health emergencies or not focused on disease prevention and control will be excluded.

## Methods and data analysis

This rapid scoping review will be conducted in accordance with the Joanna Briggs Institute (JBI) methodology for scoping reviews (9). The review will be reported using the PRISMA Extension for Scoping Reviews (PRISMA-ScR) (12) and PRISMA-S Extension for Searches in Systematic Reviews (13).

### Search strategy

The search strategy will aim to locate peer-reviewed review or empirical research (any study design) that is available in full-text and published in scientific journals, publicly available policy



For peer review only - http://bmjopen.bmj.com/site/about/guidelines.xhtml

frameworks, programme reports, and published conference reports or electronic theses. This will include humanitarian literature where health impacts or effects are the focus. Due to the rapid nature of the scoping review, we will restrict the search to studies published between 2010 – 2021 and those available in English (potentially eligible Afrikaans, German or French full texts, according to the English abstract, will be translated into English).

The electronic databases to be searched include MEDLINE, Embase and Web of Science. An initial limited search of MEDLINE was undertaken to identify articles on the review topic. The text words contained in the titles and abstracts of relevant articles, and the index terms used to describe the articles were used to draft a full search strategy for MEDLINE. The search strings and terms were developed iteratively and in consultation with the WHO and are centred around three key concepts: (i) emergencies/ diseases/ natural disasters; (ii) readiness/ preparedness/ risk/ planning; and (iii) health systems/ community. The search strategy, including all identified keywords and index terms, was subsequently adapted for Embase and Web of Science. Searches will be conducted by an expert information specialist in consultation with the review team. The reference list of all included sources of evidence will also be screened for additional studies. Reporting of the searching will be guided by the PRISMA-S Extension for Searches in Systematic Reviews (13).

### *Searching other resources*

Sources of unpublished studies/ grey literature to be searched include various targeted repositories, websites, and databases. These include global organisations (e.g., the WHO, United Nation Children's Fund [UNICEF], United Nations Office for Disaster Risk Reduction [UNDRR], United Nations International Strategy for Disaster Reduction [UNISDR], International Federation of Red Cross [IFRC], International Committee of the Red Cross [ICRC]), regional WHO offices (i.e., Southeast Asian, African, Western Pacific, Pan American, European and Eastern Mediterranean) and the European Centre for Disaster Medicine (CEMEC). Societies and organisations include the World Association for Disaster and Emergency Medicine (WADEM), Médecins Sans Frontières (MSF) and ReliefWeb. National websites include the United States Centres for Disease Control and Prevention (CDC) and Federal Emergency Management Agency (FEMA), the Robert Koch Institute (RKI), Public Health England. Lastly, Evidence Aid will be included as an evidence repository.

## Selection of studies

All search hits will be imported into Rayyan V0.1.0 software (Rayyan Systems Inc., MA, USA) (14) for screening, checking of duplicates and selection of final documents to be included. To support consistent abstract and title screening and refine eligibility, senior authors (RE, HG and MM) together with the title and abstract screeners (MP and MYC), will (as an initial step) independently and in duplicate screen 100 articles, followed by discussion. The following proposed screening approach is adapted from the Cochrane Rapid Reviews Methods Group guidance for systematic reviews to balance rigour and speed consistent with rapid reviews (15,16). Twenty percent of titles and abstracts will be screened by two reviewers (MP and MYC), independently, in duplicate and with conflict resolution, to remove obviously irrelevant reports. After this, one reviewer (MP) will



For peer review only - http://bmjopen.bmj.com/site/about/guidelines.xhtml

screen the remaining titles and abstracts while the second reviewer (MYC) will verify excluded titles and abstracts and resolve conflicts (15). If required, a third senior reviewer (HG or RE) will resolve any disagreements The full texts of selected citations will subsequently be assessed in detail against the eligibility criteria by the first reviewer, while the second reviewer will verify all excluded full texts (15). Reasons for exclusion of sources of evidence at full text that do not meet the inclusion criteria will be recorded. This information will be reported and added to a table of excluded studies in the scoping review. Any disagreements that arise between the reviewers at each stage of the selection process will be resolved through discussion, including with an additional senior reviewer (HG or RE) if needed. If study eligibility is unclear owing to missing data, further information will be requested from study authors. The results of the search and the source inclusion process will be reported in full in the final scoping review and presented in a PRISMA-ScR flow diagram (12).

## Data extraction and management

Due to the rapid design and potential large pool of included studies we will use a dynamic approach to data extraction and management. For an included study yield of ≤25, data extraction will be done by one reviewer (MP), while a second reviewer (KB or MYC) will check for completeness and accuracy (15). For yields between >25 but ≤75, two or more extractors will be used (e.g., MP/KB/MYC/CJ/QL/RE), while an additional reviewer will check for correctness and accuracy (17). In the case of more than 75 included sources, we will consider a prioritisation process whereby we rank or stratify studies based on design and relevancy to the scoping review. Prioritised studies will then be included for data extraction until the review team, together with WHO, agrees that data saturation has been achieved. The reviewers will discuss the nature of the information that will be extracted before commencing the process to facilitate coherence. Any uncertainties before and during the extraction process will be discussed with team members to make a final decision.

The data extracted will include author name(s), publication year, publication country and World Bank classification, source classification as primary/ secondary/ multi-method, publication type, study design, aim/ purpose, sample/ facility description, method/ tool for data collection, modifications to the data tool (if any), level (community, national, etc.), type of emergency, operational readiness definition, preparedness definition, key actors, challenges/ recommendations, lessons learnt, and other relevant information/ conclusions. In addition, data regarding readiness will be extracted according to the WHO's operational readiness components – these include:

- Leadership, governance, and coordination,
- Country risk profile,
- Operational planning and coordination,
- Contingency finance,
- Health facility capacity and service delivery,
- Health workforce/ human resources,
- Early warning or surveillance and health information systems,



For peer review only - http://bmjopen.bmj.com/site/about/guidelines.xhtml

- Community resilience and risk communications,
- Logistics or supply chain for access to essential medicines,
- WHO readiness,
- Partner readiness.

Framework details and any associated actions will be recorded. Finally, information regarding relevant models will be extracted, including URL links to figures/ diagrams.

A draft extraction form will be pilot-tested independently by two reviewers using a sample of two to three potential included full-text articles/ evidence sources (17). Based on feedback from the two reviewers, the form may be modified and revised as necessary during the process of extracting data from each included evidence source (17). Necessary modifications will first be discussed within the review team for consensus, and any changes implemented will be reported in the final scoping review. Authors will be contacted where possible to clarify or obtain additional information.

## Methodological appraisal

Included peer-reviewed literature will be evaluated for quality based on appropriate pre-existing methodological quality checklists.

## Data analysis and presentation

Data will be synthesised in line with the core objectives of the rapid scoping review.

The included documents will be analysed using qualitative thematic analysis through an deductive synthesis approach (18–20). We are proposing to use ATLAS.ti V8 (Scientific Software Development GmbH) (https://atlasti.com/) to conduct thematic data analysis as well as store, organise, and retrieve data. Data analysis will be carried out by the project group researchers, who have vast knowledge and experience in undertaking reviews, including scoping reviews, that have used qualitative thematic analysis.

Findings will be deductively coded into a conceptual model that is taken from the WHO Country Readiness for Health Checklist to define and identify the critical elements of 'operational readiness' for public health emergencies, including COVID-19, and identify lessons learnt from addressing it. We will also identify if there are additional consistent themes emerging from the analysis that are not currently included in the WHO Checklist, as potential additional items.

The analysis will start by evaluating documented text line-by-line, allocating text a descriptive label and code. The same will be done for the other focused questions on understanding the similarities and differences between operational readiness and preparedness and identifying critical elements. The researchers will remain close to the data from the primary sources when defining and understanding the meaning structure of these concepts and phenomena. Since the conceptual understanding of 'operational readiness' and 'preparedness' will be initially explored, described,



For peer review only - http://bmjopen.bmj.com/site/about/guidelines.xhtml

and theorised and may vary across sources, we will initially use broad, higher order codes (which may form main themes) developed deductively from the framework to organise the data. Once all data have been initially coded and collated, all the potentially relevant coded data extracts will be sorted and collated into themes and sub-themes (including a 'miscellaneous' theme for codes that do not clearly fit into existing themes (20). Senior reviewers (RE, HG and QL) will debrief the researchers primarily responsible for the thematic analysis, and the review team will meet regularly to discuss codes and themes, including potential merging or further break-down of themes (depending on whether there are enough data to support a theme, or the data are deemed too diverse). The themes will represent the synthesis and interpretation that go beyond the primary sources as well as deliver new insights and knowledge, which will translate into an operational framework for readiness and important lessons learnt.

A numerical description of the extent and nature of included evidence sources will be presented using tables and charts, accompanied by narrative summaries to describe how the results relate to the review's objectives.

## Patient Public involvement

Patients or the public were not involved in the design, or conduct, or reporting, or dissemination plans of our research

## Ethics, reporting and dissemination

No ethical approval is needed for this rapid scoping review, given that included sources will comprise of published and publicly available information.

The study was expected to commerce in December 2021 to July 2022 with first scientific publication output expected in August 2022. The Stellenbosch University (SU) review team will work with the WHO commissioning group and draw on the expertise of expert advisors to the review team to produce the following outputs. Weekly internal and SU-WHO meetings have been conducted to provide input into the development of this research protocol and will continue to aid understanding of emerging insights and findings that can inform work tasks relevant to the technical product development. Interim findings from the rapid scoping review will be presented to the WHO. Following feedback, an updated interim report incorporating feedback from the WHO and expert advisory team will be presented. The final report of the full rapid scoping review will be delivered, along with a PowerPoint presentation to the WHO commissioning group of findings with talking points. In consultation with the WHO, findings will be disseminated further as appropriate (e.g., through professional bodies, conferences, and research papers). By defining evidence related to critical readiness components and actions, this review will reveal new insights, knowledge and lessons learnt that will translate into an operational framework for readiness actions.



For peer review only - http://bmjopen.bmj.com/site/about/guidelines.xhtml

# Acknowledgements

The rapid scoping review was commissioned by the WHO to inform an Operational Readiness Framework for the Country Readiness Strengthening Department in the World Health Emergencies Program in WHO (Reference #: 2021/1145765; Unit: MST; Cluster: QNF/SCI).


# Authors' contribution

RE is the Principal investigator and JCYN coordinates the team, the research process and is a corresponding author. NG and LB contributed to content through the process, provided guidance on the expected outcomes and WHO guidelines to be followed. KB drafted the original protocol, MM, QL, CJ and HG provided technical guidance on the methodology. All authors were involved in the conceptualization of the concept paper and proposal writing and read the final version of the submitted protocol.


# Funding

This work is supported by the World Health Organization through a consultancy fee and not research grant [Reference: APW/RR/Readiness/2021/1145765], as a result, there is no grant number


# Conflicts of interest

There is no conflict of interest in this project.

For peer review only



For peer review only - http://bmjopen.bmj.com/site/about/guidelines.xhtml

## References


1. Savoia E, Lin L, Viswanath K. Communications in public health emergency preparedness: A systematic review of the literature [Internet]. Vol. 11, Biosecurity and Bioterrorism. Mary Ann Liebert, Inc.; 2013 [cited 2021 Oct 4]. p. 170–84. Available from: /pmc/articles/PMC3778998/

2. Tang R, Fitzgerald G, Hou XY, Wu YP. Building an evaluation instrument for china's hospital emergency preparedness: A systematic review of preparedness instruments [Internet]. Vol. 8, Disaster Medicine and Public Health Preparedness. Lippincott Williams and Wilkins; 2014 [cited 2021 Oct 4]. p. 101–9. Available from: https://pubmed.ncbi.nlm.nih.gov/24622400/

3. Ejeta LT, Ardalan A, Paton D. Application of behavioral theories to disaster and emergency health preparedness: A systematic review. PLoS Curr. 2015 Jul;7(DISASTERS).

4. WHO Emergencies Preparedness Team. Emergency response framework [Internet]. 2nd ed. World Health Organization, editor. Geneva; 2017. Available from: https://www.who.int/publications/i/item/9789241512299

5. Lo STT, Chan EYY, Chan GKW, Murray V, Abrahams J, Ardalan A, et al. Health Emergency and Disaster Risk Management (Health-EDRM): Developing the Research Field within the Sendai Framework Paradigm. Int J Disaster Risk Sci [Internet]. 2017 Jun 1 [cited 2021 Oct 4];8(2):145–9. Available from: www.ijdrs.comwww.springer.com/13753

6. WHO Emergencies Preparedness Team. A strategic framework for emergency preparedness [Internet]. World Health Organization, editor. Geneva; 2016. Available from: https://www.who.int/publications/i/item/a-strategic-framework-for-emergency-preparedness

7. Haldane V, De Foo C, Abdalla SM, Jung AS, Tan M, Wu S, et al. Health systems resilience in managing the COVID-19 pandemic: lessons from 28 countries [Internet]. Vol. 27, Nature Medicine. Nature Research; 2021 [cited 2021 Oct 4]. p. 964–80. Available from: https://pubmed.ncbi.nlm.nih.gov/34002090/

8. Khan Y, O'Sullivan T, Brown A, Tracey S, Gibson J, Généreux M, et al. Public health emergency preparedness: A framework to promote resilience. BMC Public Health [Internet]. 2018 Dec 5 [cited 2021 Oct 4];18:1344. Available from: https://bmcpublichealth.biomedcentral.com/articles/10.1186/s12889-018-6250-7

9. Peters M, Godfrey C, McInerey P, Munn Z, Tricco A, Khalil H. Chapter 11: Scoping Reviews (2020 version). In: Aromataris E, Munn Z, editors. JBI Manual for Evidence Synthesis [Internet]. JBI; 2020. Available from: https://synthesismanual.jbi.global

10. Nelson C, Lurie N, Wasserman J, Zakowski S. Conceptualizing and defining public health emergency preparedness. Vol. 97 Suppl 1, American journal of public health. American Public Health Association; 2007. p. S9–11.

11. World Health Organization. Chapter 11: Public health emergencies. In: Advancing the right to health: the vital role of law [Internet]. Geneva: World Health Organization; 2017. Available from: https://apps.who.int/iris/handle/10665/252815




For peer review only - http://bmjopen.bmj.com/site/about/guidelines.xhtml

12. Tricco AC, Lillie E, Zarin W, O'Brien KK, Colquhoun H, Levac D, et al. PRISMA Extension for Scoping Reviews (PRISMA-ScR): Checklist and Explanation. Ann Intern Med [Internet]. 2018 Oct 2 [cited 2019 Apr 8];169(7):467–74. Available from: http://annals.org/article.aspx?doi=10.7326/M18-0850

13. Rethlefsen ML, Kirtley S, Waffenschmidt S, Ayala AP, Moher D, Page MJ, et al. PRISMA-S: an extension to the PRISMA Statement for Reporting Literature Searches in Systematic Reviews. Syst Rev [Internet]. 2021 Jan 26 [cited 2021 Oct 4];10(1):39. Available from: https://doi.org/10.1186/s13643-020-01542-z

14. Ouzzani M, Hammady H, Fedorowicz Z, Elmagarmid A. Rayyan -a web and mobile app for systematic reviews. Syst Rev [Internet]. 2016 [cited 2021 Oct 4];5(1):210. Available from: http://systematicreviewsjournal.biomedcentral.com/articles/10.1186/s13643-016-0384-4

15. Garritty C, Gartlehner G, Nussbaumer-Streit B, King VJ, Hamel C, Kamel C, et al. Cochrane Rapid Reviews Methods Group offers evidence-informed guidance to conduct rapid reviews. J Clin Epidemiol [Internet]. 2021 Feb 1 [cited 2021 Oct 4];130:13–22. Available from: https://doi.org/10.1016/j.jclinepi.2020.10.007

16. Tricco AC, Garritty CM, Boulos L, Lockwood C, Wilson M, McGowan J, et al. Rapid review methods more challenging during COVID-19: commentary with a focus on 8 knowledge synthesis steps [Internet]. Vol. 126, Journal of Clinical Epidemiology. Elsevier Inc.; 2020 [cited 2021 Oct 6]. p. 177–83. Available from: http://www.jclinepi.com/article/S0895435620306168/fulltext

17. Peters MDJ, Marnie C, Tricco AC, Pollock D, Munn Z, Alexander L, et al. Updated methodological guidance for the conduct of scoping reviews. JBI Evid Synth [Internet]. 2020 Oct 1 [cited 2021 Oct 6];18(10):2119–26. Available from: https://journals.lww.com/10.11124/JBIES-20-00167

18. Thomas J, Harden A. Methods for the thematic synthesis of qualitative research in systematic reviews. BMC Med Res Methodol [Internet]. 2008 Dec 10 [cited 2021 Oct 5];8(1):45. Available from: https://bmcmedresmethodol.biomedcentral.com/articles/10.1186/1471-2288-8-45

19. Levac D, Colquhoun H, O'Brien KK. Scoping studies: Advancing the methodology. Implement Sci [Internet]. 2010 Sep 20 [cited 2021 Oct 5];5(1):69. Available from: http://implementationscience.biomedcentral.com/articles/10.1186/1748-5908-5-69

20. Nowell LS, Norris JM, White DE, Moules NJ. Thematic Analysis: Striving to Meet the Trustworthiness Criteria. Int J Qual Methods [Internet]. 2017 Dec 2 [cited 2021 Oct 13];16(1):160940691773384. Available from: http://journals.sagepub.com/doi/10.1177/1609406917733847



For peer review only - http://bmjopen.bmj.com/site/about/guidelines.xhtml
